# Increased expression of BRD4 isoforms long (BRD4-L) and short (BRD4-S) promotes chemotherapy resistance in high-grade serous ovarian carcinoma

**DOI:** 10.18632/genesandcancer.233

**Published:** 2023-09-12

**Authors:** Ana Luiza Drumond-Bock, Luyao Wang, Lin Wang, Magdalena Cybula, Maria Rostworowska, Michael Kinter, Magdalena Bieniasz

**Affiliations:** ^1^Aging and Metabolism Research Program, Oklahoma Medical Research Foundation, Oklahoma City, OK 73104, USA; ^2^Cytovance Biologics, Oklahoma City, OK 73104, USA

**Keywords:** high-grade serous ovarian carcinoma, BRD4 amplification, BRD4 long, BRD4 short, chemoresistance

## Abstract

Chemoresistance in ovarian carcinoma is a puzzling issue that urges understanding of strategies used by cancer cells to survive DNA damage and to escape cell death. Expanding efforts to understand mechanisms driving chemoresistance and to develop alternative therapies targeting chemoresistant tumors are critical. Amplification of *BRD4* is frequently associated with chemoresistant ovarian carcinoma, but little is known about the biological effects of the overexpression of BRD4 isoforms in this malignancy. Here, we described the consequences of BRD4-L and BRD4-S overexpression in ovarian carcinoma shedding a light on a complex regulation of BRD4 isoforms. We demonstrated that the BRD4-L transcript expression is required to generate both isoforms, BRD4-L and BRD4-S. We showed that the BRD4-S mRNA expression positively correlated with BRD4-S protein levels, while BRD4-L isoform showed negative correlation between mRNA and protein levels. Moreover, we demonstrated that an overexpression of BRD4 isoforms is associated with chemoresistance in ovarian cancer.

## INTRODUCTION

High-Grade Serous Ovarian Carcinoma (HGSOC) is the most prevalent type of ovarian cancer [1], and one of the deadliest gynecological malignancies in the United States [2, 3]. The treatment of patients with HGSOC becomes particularly challenging because the disease is often diagnosed in advanced stages [1], and recurrence of malignant cancer commonly occurs within 2 years after initial therapy in the majority of patients [4]. Due to the aggressive disease profile and the poor overall patients’ survival, HGSOC remains a major unmet clinical challenge and is one of the most investigated types of gynecological cancers.

Studies performed by The Cancer Genome Atlas (TCGA) initiative have provided scientists with a broad overview of the genetic events happening in a variety of cancers, including HGSOC [5]. One of the most common features amongst ovarian carcinoma patients is the loss of *TP53* function due to genetic mutations occurring in 96% of the patients. In addition, HGSOC shows a high level of genomic instability [6] associated with increased somatic copy number alterations, a result of deletion or amplification of a large number of genes [5]. Among the top 5 most amplified genes in HGSOC is *BRD4*, which is amplified in 18% of patients’ tumors [7]. Amplification of *BRD4* in ovarian tumors correlates with worse disease-free progression and overall survival of patients. In addition, *BRD4* amplification has been recognized as a major contributing factor in chemotherapy resistance and treatment failure [8–10]. Due to these findings, BRD4 has gained a growing interest in studies exploring mechanisms of HGSOC progression [7, 9, 11–13], and became a promising therapeutic target under preclinical investigation [14, 15].

The *BRD4* gene encodes several isoforms of BRD4 protein [16], which are generated by alternative splicing and are constitutively expressed. The two most common isoforms of BRD4 are the long isoform BRD4-L and the short isoform BRD4-S(a) (hereafter BRD4-S(a) is abbreviated as BRD4-S) [16, 17]. These two isoforms contain identical N-terminal segments [18, 19] encompassing two tandem bromodomains, however BRD4-L contains an extra C-terminal domain that is needed for its function as a transcriptional co-activator [20], while BRD4-S contains three additional N-terminal residues referred as GPA (glycine-proline-alanine) [19]. In ovarian cancer patients, amplification of *BRD4* correlates with the overexpression of BRD4-L and BRD4-S mRNAs [7], as well as the overexpression of BRD4 proteins. Little is known about the consequential events involved in the increased expression of BRD4 isoforms in those patients, however, it has been reported that the overexpression of BRD4-S in immortalized ovarian surface epithelial cells is capable of inducing oncogenic transformation of these cells [7].

Analysis of BRD4 isoforms in malignancies other than ovarian carcinoma has suggested that BRD4 isoforms play distinct roles in carcinogenesis [21–26]. Early studies have defined the tumor-protective function of BRD4 in breast cancer and Hutchinson-Gilford progeria syndrome [23, 25, 26]. Alsarraj and colleagues have reported that the ectopic expression of BRD4-S in breast cancer increases its metastatic potential, while the overexpression of BRD4-L suppresses tumor growth and occurrence of metastasis [23]. In addition, a recent study using breast cancer cell lines has provided detailed molecular mechanisms illustrating how BRD4-S promotes oncogenic transformation and the mechanism by which BRD4-L exhibits tumor-suppressor functions [21]. Although it would be plausible to assume that a similar mechanism is at play in HGSOC, no study to date has provided information on the opposite roles of BRD4-S and BRD4-L in ovarian carcinomas. Furthermore, although BRD4-S is frequently defined as a tumor-promoting protein, while BRD4-L is described as a tumor-suppressor based on studies evaluating tumor growth and metastatic potential [9, 21, 23, 25, 26], there is scarcity of studies interrogating the response to chemotherapy of tumors overexpressing respective BRD4 isoforms. The response to platinum-taxane chemotherapy in the context of aberrantly activated BRD4 isoform(s) is a highly relevant area of research considering the poor prognosis of ovarian carcinoma patients harboring *BRD4* amplification. Our lab is interested in addressing this gap of knowledge, which will advance our understanding of chemotherapy resistance mechanisms and could reveal new therapeutic options for ovarian cancer patients.

Since the *BRD4* amplification is frequently associated with chemoresistant-disease in HGSOC patients [8–10, 27], it is possible that the overexpression of BRD4 isoforms could be an essential factor contributing to resistance to DNA-damaging agents, such as platinum-based drugs. Platinum-resistant ovarian carcinoma cells show an increased activity of DNA damage repair (DDR), which is the major mechanism of resistance to this treatment. Instead of undergoing cell death due to an extensive DNA damage induced by platinum drugs, the platinum-resistant cancer cells enhance their DNA damage repair mechanisms in order to survive [28]. Previously published data showed that as a transcriptional cofactor, BRD4 promotes DNA repair by regulating activation of genes involved in DDR, such as aldehyde dehydrogenase and CHK1 [29–32]. Furthermore, there is evidence that BRD4-S impairs DNA repair by shielding the chromatin from the proteins involved in DDR signaling [33]. Nevertheless, further empirical studies are needed to establish the underlying mechanism by which the overexpression of BRD4 isoforms contributes to development of chemotherapy resistance.

Hence, in the present work, we provide a broader understanding of the consequences of BRD4-L and BRD4-S overexpression in HGSOC, as well as an integrated insight into the response of BRD4-amplified tumors to first-line chemotherapy. Our preclinical *in vivo* studies with the use patient-derived xenografts (PDXs), demonstrated that the increased expression of both isoforms, BRD4-L and BRD4-S is associated with an increased resistance to standard-care chemotherapy (cisplatin/paclitaxel). Finally, we observed that the combination of paclitaxel with selected targeted therapies shows potent antitumor efficacy *in vitro*, and represents a much needed new opportunity for therapeutic intervention in HGSOC patients with *BRD4* amplification.

## RESULTS

### Overexpression or knockdown of BRD4 isoforms induce their distinct transcription patterns

**Figure 1:**
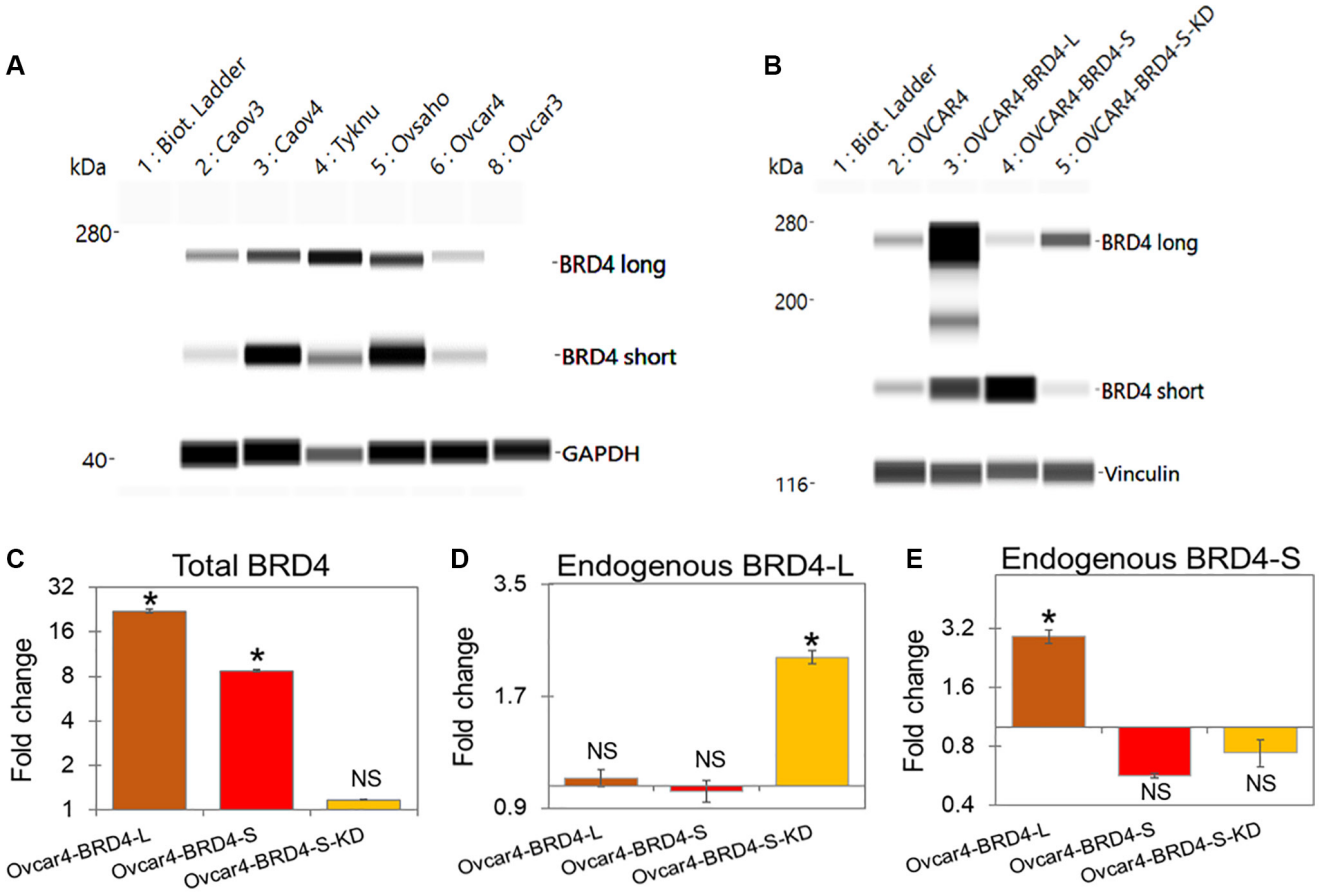
Overexpression or knockdown of BRD4 isoforms induce their distinct transcription patterns. (**A**) Wes (ProteinSimple) showing protein expression of BRD4 long (BRD4-L) and BRD4 short (BRD4-S) in ovarian cancer cell lines representing high-grade serous ovarian carcinoma. (**B**) Protein expression assessed by Wes of BRD4 isoforms in Ovcar4 cell lines following BRD4 isoforms overexpression or knockdown (KD). The expression of housekeeping proteins GAPDH and vinculin was used as loading control. Whole Wes images are included in Supplementary Figures 1, 2. (**C**–**E**) Relative expression of transcripts encoding BRD4 isoforms in Ovcar4 cell lines. (C) Total (endogenous and exogenous) levels of BRD4 mRNA. (D) Endogenous levels of BRD4-L mRNA. (E) Endogenous levels of BRD4-S mRNA. Fold change of BRD4 isoform expression in genetically engineered Ovcar4 cell lines is presented as relative to the expression of respective BRD4 isoform in control (parental Ovcar4 cells). Results are presented as average ± SE, ^*^p < 0.05, NS indicate not statistically significant.

In order to evaluate the expression levels of BRD4 isoforms in HGSOC, we performed Wes (ProteinSimple) analysis using a panel of human ovarian carcinoma (OC) cell lines (Figure 1A). Wes from ProteinSimple is an automated capillary-based immunoassay used to detect and quantify proteins, where results are presented as Wes images resembling a traditional Western Blot data. Based on BRD4 protein levels quantification analysis by Wes, we selected Ovcar3 and Ovcar4 cell lines with low and average protein levels of both isoforms, respectively (Figure 1A and Supplementary Figure 1) and overexpressed BRD4-L or BRD4-S isoforms in these cells using lentiviral vectors (Figure 1B and Supplementary Figures 2A–2C and 3A). Since Ovcar3-BRD4-S cells failed to grow *in vitro* (data not shown), thus, further studies were carried out with the use of Ovcar4 cell line. Next, we performed a real-time RT-PCR to quantify the relative transcript levels of BRD4 isoforms in these cell lines (Figure 1C–1E). We designed three sets of qPCR primers. First set of primers was used to analyze changes in total levels of BRD4 transcripts reflecting combined expression of BRD4-L and BRD4-S mRNAs. The remaining primers were designed to detect only endogenous levels of either BRD4-L or BRD4-S mRNA (the exogenous BRD4 transcripts introduced by lentiviral overexpression vectors were not detected by these primers). This approach allowed us to investigate if changes in the expression levels of BRD4-L isoform affect the expression of BRD4-S and vice versa. We observed a significant increase of total BRD4 mRNA in Ovcar4-BRD4-L and Ovcar4-BRD4-S cell lines, as expected (Figure 1C), which correlated with increased protein levels of BRD4 isoforms assessed by Wes (Figure 1B), and by mass spectrometry (Table 1). We also observed that the overexpression of BRD4-L correlated with increased expression of BRD4-S transcript (Figure 1E), while knockdown of BRD4-S isoform was consistent with increased expression of BRD4-L mRNA (Figure 1D). These changes in transcript levels were followed by similar changes in the BRD4 protein expression (Table 1). Taken together, our results suggest that BRD4-L mRNA is required to generate both isoforms, and that the depletion of BRD4-S isoform is associated with increased expression of BRD4 long isoform.

**Table 1:** Protein quantification of BRD4 isoforms by mass spectrometry

Cell Line	Total BRD4 (pmol/mg)	BRD4-L (pmol/mg)	Other BRD4 isoforms (pmol/mg)
Ovcar4	0.46	0.05	0.41
Ovcar4-BRD4-L	3.55^*^	0.57^*^	2.98^*^
Ovcar4-BRD4-S	2.78^*^	0.04	2.74^*^
Ovcar4-BRD4-S-KD	0.37^*^	0.13^*^	0.24^*^

Table represents quantification of BRD4-L specific peptides and total BRD4 peptides. Quantification of other BRD4 isoforms (including BRD4-S) was estimated by extracting the amount of BRD4-L from total BRD4. Data are expressed as average ± SE. Four replicates were tested for each cell line. ^*^p < 0.05.

### Overexpression of distinct BRD4 isoforms has opposing roles in promoting ovarian cancer cell proliferation *in vitro*

**Figure 2:**
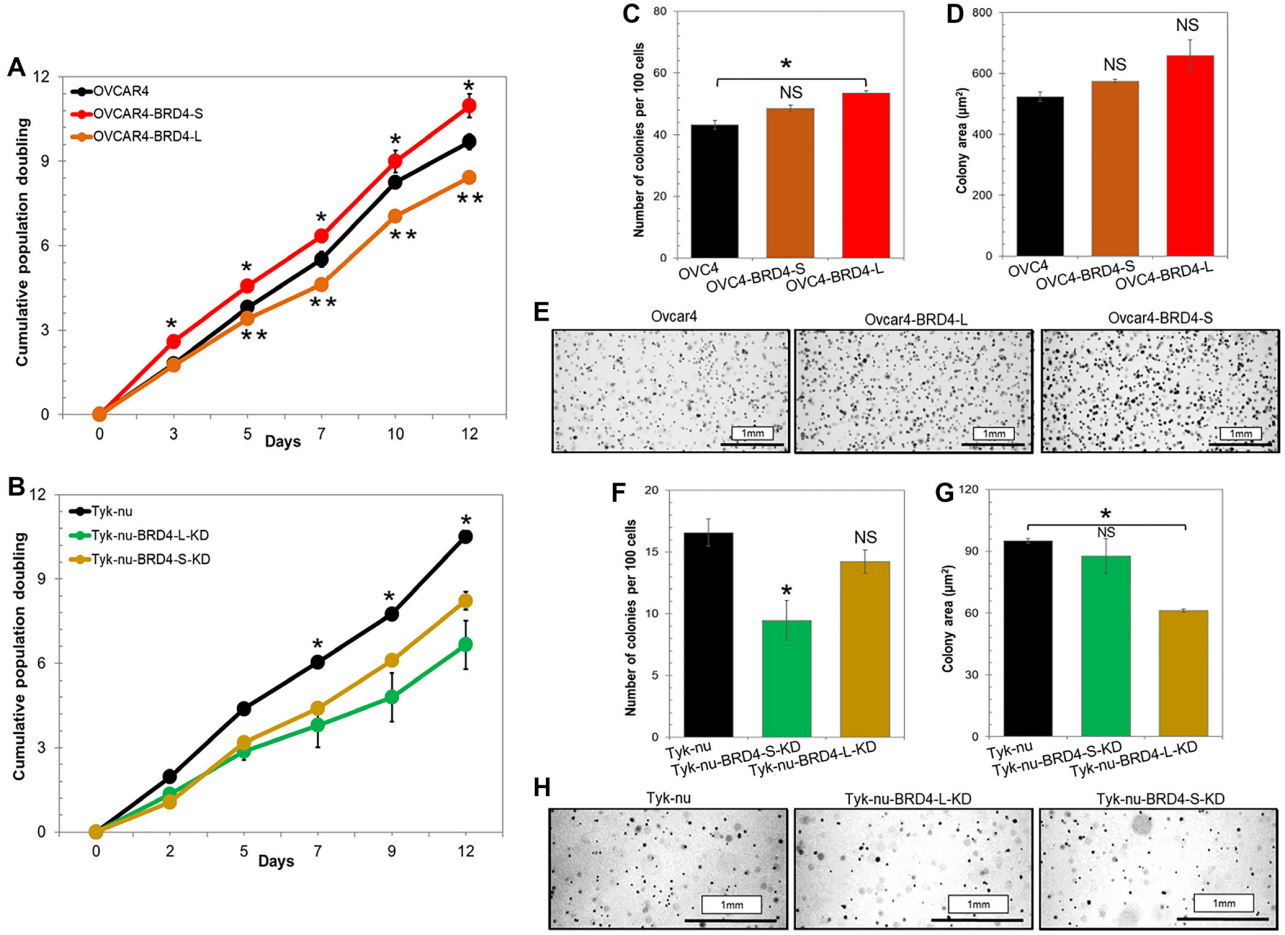
Overexpression of distinct BRD4 isoforms has opposing roles in promoting ovarian cancer cell proliferation *in vitro*. (**A**) Cumulative cell population doubling quantified by 3T5 cell proliferation assay of Ovcar4 cells overexpressing BRD4-L or BRD4-S compared to control cells. (**B**) Cell proliferation rate of Tyk-nu cells following BRD4-L or BRD4-S knockdown compared to parental cells. Results are represented as average ± SD; ^*^p < 0.05, and ^**^p < 0.01 (one-way ANOVA followed by Tukey’s post-hoc). (**C**–**E**) Anchorage-independent growth of Ovcar4 cells overexpressing individual BRD4 isoforms was assessed by colony formation assay (soft agar colony formation assay). Number of colonies was quantified in (C) and their size in was measured in (D). (E) Representative images of cell colonies grown in soft agar captured at day 11. (**F**–**H**) Anchorage-independent growth of Tyk-nu cells following BRD4 isoform knockdown. Number of colonies was quantified in (F) and their size in was measured in (G). (H) Representative images of cell colonies grown in soft agar captured at day 11. Results are presented as average ± SE, ^*^p < 0.05, NS not significant when compared to control (unpaired *t*-test). More detailed colony formation assay data are provided in Supplementary Figure 4.

We investigated the effects of overexpression or knockdown of individual BRD4 isoforms on ovarian cancer cell proliferation *in vitro*. Cell population doubling assay demonstrated that distinct BRD4 isoforms such as BRD4-L or BRD4-S have opposing roles in promoting cell proliferation. Ovcar4-BRD4-S cells proliferated considerably faster than the parental Ovcar4, while Ovcar4-BRD4-L showed slower cell proliferation rate (Figure 2A). Slower cell proliferation rate was also observed in Ovcar3-BRD4-L, when compared to parental Ovcar3 cells (Supplementary Figure 3B). Further, to assess the effect of BRD4 isoform knockdown on cell proliferation and colony formation in anchorage-independent conditions, we performed a stable knockdown of BRD4 isoforms in Tyk-nu cell line that expresses high levels of both isoforms (Supplementary Figure 2D–2G). We observed that both Tyk-nu-BRD4-L-KD and Tyk-nu-BRD4-S-KD proliferated significantly slower than parental Tyk-nu cells (Figure 2B). The anchorage-independent growth assay revealed that Ovcar4 cells overexpressing BRD4-S produced a higher number of cell colonies in soft agar, which tended to be larger than the colonies produced by parental Ovcar4 cell line (Figure 2C–2E and Supplementary Figure 4). In contrast, the overexpression of BRD4-L isoform has either no effect on number and size of colonies in Ovcar4 cell line (Figure 2C, 2D) or leads to generation of less colonies in Ovcar3 cells when compared with respective controls (Supplementary Figure 3B–3E). Further, a depletion of BRD4-L isoform in Tyk-nu cells resulted in generation of considerably fewer colonies when compared with parental Tyk-nu cells or those with BRD4-S knockdown (Figure 2F, 2H and Supplementary Figure 4). In contrast, we observed no change in number of cell colonies between Tyk-nu and Tyk-nu-BRD4-S-KD cell lines, while the BRD4-S depleted cells generated smaller colonies (Figure 2G, 2H and Supplementary Figure 4).

In summary, our findings demonstrated that the overexpression of individual BRD4 isoforms has a distinct impact on cellular phenotype. The overexpression of BRD4-L isoform is associated with reduced cell proliferation. In contrast, the overexpression of BRD4-S is associated with a significantly faster cell proliferation and an improved ability of cells to survive and generate colonies in anchorage-independent conditions, which is consistent with a more aggressive tumor phenotype.

### Ovarian carcinoma cells overexpressing BRD4-S isoform become arrested in G2/M phase of the cell cycle

**Figure 3:**
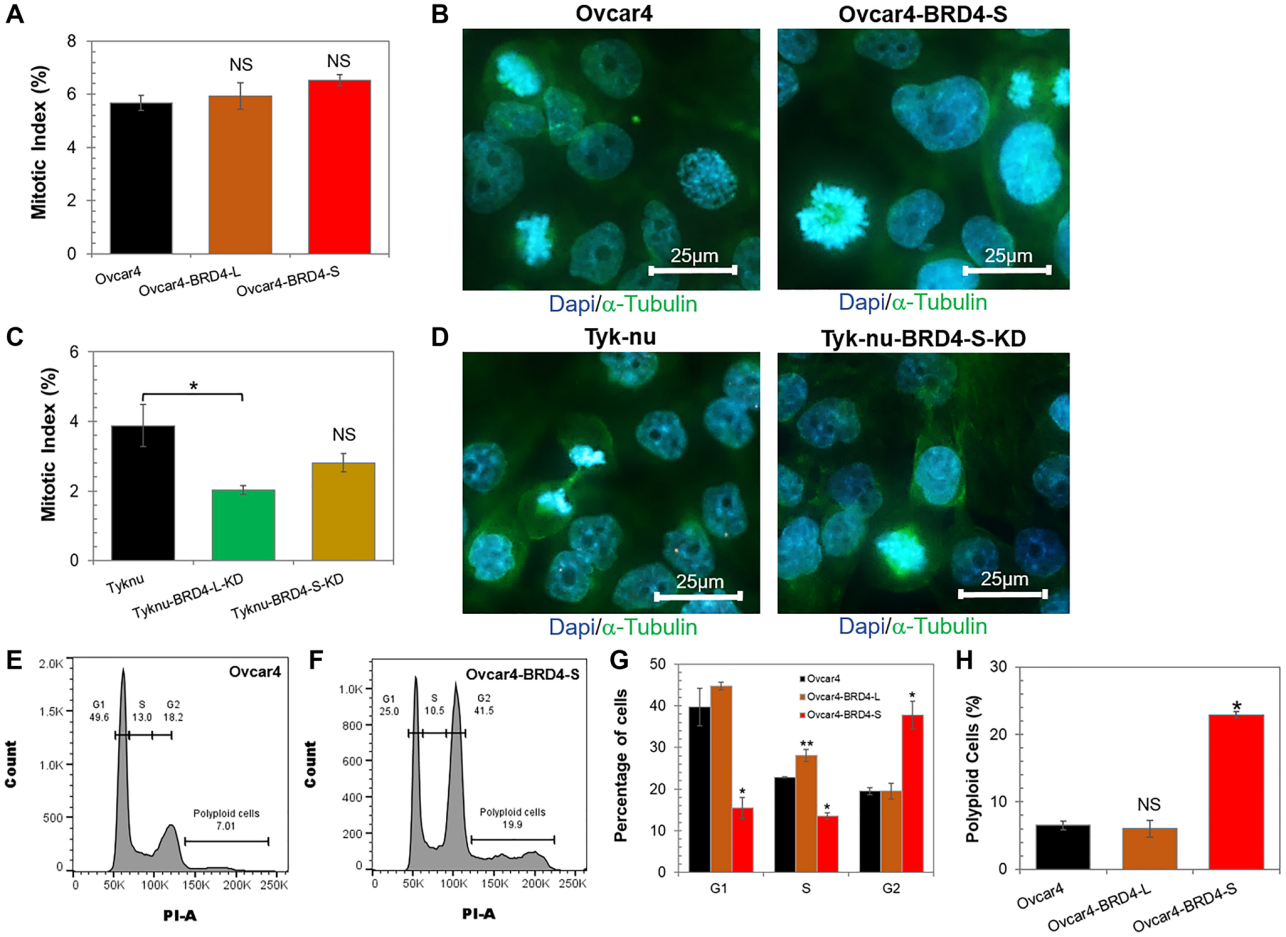
Ovarian carcinoma cells overexpressing BRD4-S isoform become arrested in G2/M phase of the cell cycle. (**A**) Mitotic Index (MI) defined as the percentage of cells undergoing mitosis in a given population of cells was quantified for Ovcar4 cell lines overexpressing BRD4 isoforms vs. Ovcar4 control cells. Results are presented as average ± SE, NS - not significant when compared to control (unpaired *t*-test). (**B**) Images of mitotic cells detected in Ovcar4 and Ovcar4-BRD4-S cell lines. Immunofluorescence staining for α-tubulin (green) shows mitotic spindles, and DAPI staining (blue) shows chromatin conformation in different phases of mitotic cell division. (**C**) Percentage of cells in mitosis among Tyk-nu cell lines depleted of respective BRD4 isoforms vs. parental control cells. Results are presented as average ± SE, ^*^p < 0.05, NS - not significant when compared to control (unpaired *t*-test). (**D**) Images of mitotic cells detected in Tyk-nu and Tyk-nu-BRD4-S-KD cell lines. (**E**, **F**) Flow cytometry analysis of cell cycle distribution in Ovcar4 cells (E) and Ovcar4-BRD4-S cells (F). (**G**) Quantification of cells in different cell cycle phases. Results are presented as average ± SE, ^*^p < 0.05. Statistical significance was evaluated by comparing the % of cells in a given cell cycle phase between BRD4 isoform overexpressing cell line vs. control (parental) cell line (one-way ANOVA followed by Tukey’s post-hoc). (**H**) Percentage of polyploid giant cells in Ovcar4 cell lines. Data are presented as average ± SE, ^*^p < 0.05, ^**^p < 0.01, NS - not significant when compared to control (unpaired *t*-test).

We observed significant changes in cell proliferation rate following the overexpression of individual BRD4 isoforms, which prompted us to investigate the cell cycle function in those cells. The analysis of mitotic index (MI) in Ovcar4 cell lines (Figure 3A) revealed that regardless of a type of the BRD4 isoform overexpressed, there were no significant differences in the number of cells entering mitosis or quality of mitotic cells at a given time (Figure 3A, 3B). We noticed a tendency towards increased MI in Ovcar4-BRD4-S cells (Figure 3A). Subsequent flow cytometry analysis of the cell cycle distribution (Figure 3E–3G and Supplementary Figure 5) revealed that Ovcar4-BRD4-S cells show almost 2-fold higher number of cells arrested in G2/M phase than Ovcar4 and Ovcar4-BRD4-L. In addition, we observed that the overexpression of BRD4-S in Ovcar4 cells results in mitotic defects associated with generation of polyploidy cells. We detected an average of 23% of polyploidy cells in Ovcar4-BRD4-S cells vs. 6% of these cells in Ovcar4 or Ovcar4-BRD4-L (Figure 3H). The analysis of Tyk-nu cell lines revealed a reduced MI following the knockdown of either of the two BRD4 isoforms (Figure 3C, 3D), which is consistent with considerably slower cell proliferation rate of these cells (as shown in Figure 2B). However, the cell cycle function in Tyk-nu cells appear to be not affected by BRD4-S or BRD4-L knockdown (Supplementary Figure 5).

Overall, the most interesting finding from these studies was the observation that BRD4-S is the only BRD4 isoform, which strongly induces cell cycle arrest in G2/M phase and generation of polyploid cells. This cell phenotype is consistent with cells undergoing cellular stress associated with DNA damage that often leads to the G2/M cell cycle arrest. If cellular stress is prolonged, the arrested cells can resume cell proliferation even in a presence of unresolved DNA defects leading to generation of polyploid cells [34], which are the features observed in Ovcar4-BRD4-S cells.

### The BRD4-S overexpression or knockdown increases DNA damage in ovarian carcinoma cell lines

**Figure 4:**
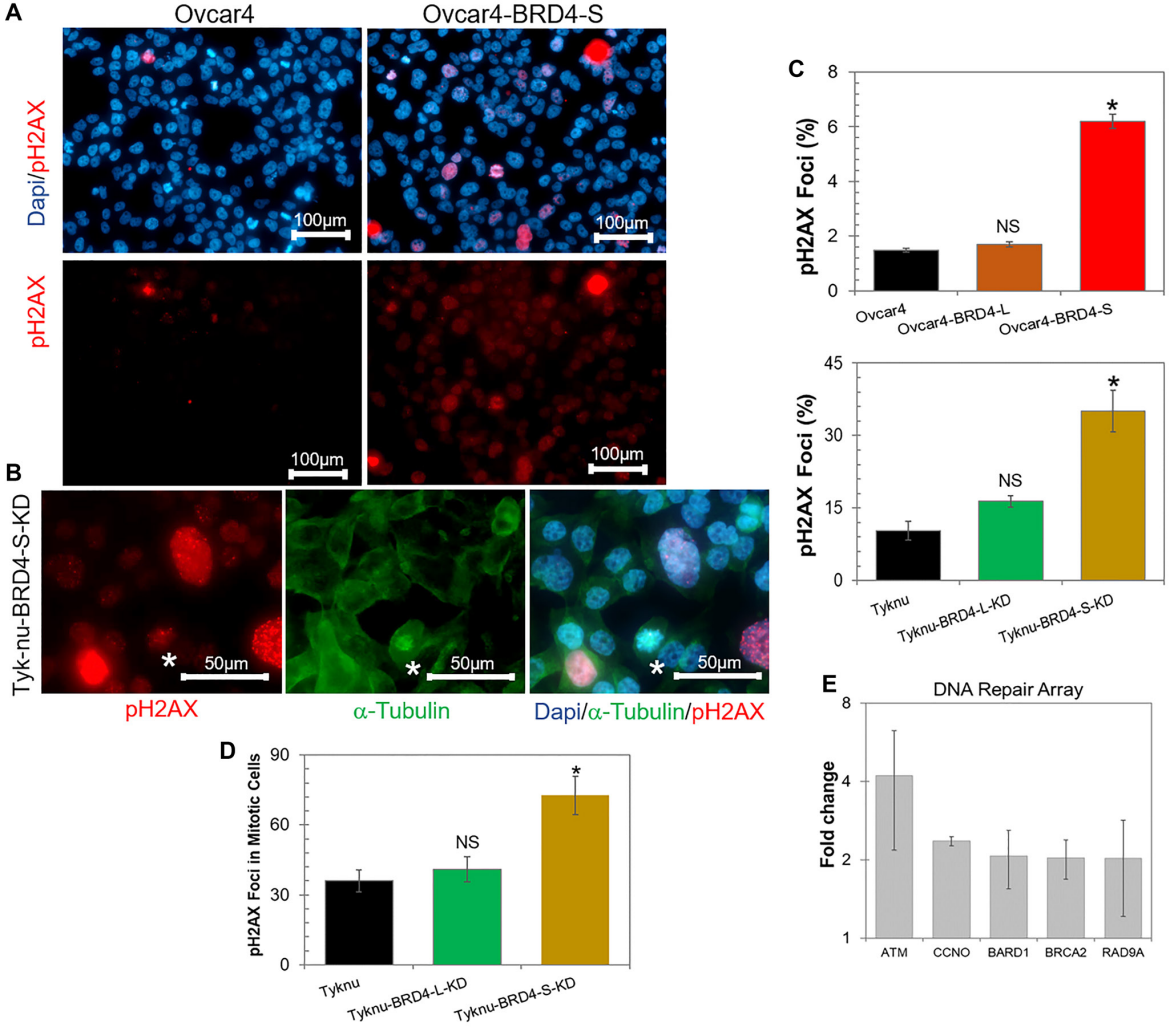
The BRD4-S overexpression or knockdown increases DNA damage in ovarian carcinoma cell lines. (**A**) Representative images of Ovcar4 and Ovcar4-BRD4-S cell lines assessed for presence of DNA damage by quantification of a number of cells with pH2AX foci. Immunofluorescence staining identified phosphorylated histone H2AX (pH2AX) foci (red) indicating cells with DNA damage. The cells were counterstained with DAPI (blue) as a chromatin marker for nucleus visualization. (**B**) Representative images of Tyk-nu-BRD4-S-KD cell line showing DNA damage (pH2AX, red) in a cell undergoing mitosis (white star). Mitotic spindles were visualized by α-tubulin staining (green), and cell nuclei were visualized by DAPI staining (blue). (**C**) Quantification of the percentage of cells with more than 20 pH2AX foci in cell lines with overexpression or knockdown of BRD4 isoforms vs. parental cells. Data expressed as average ± SE; ^*^p < 0.05, NS - not significant when compared to control (unpaired *t*-test). (**D**) Quantification of mitotic cells with pH2AX foci (as those shown in B) in Tyk-nu cell lines depleted of BRD4 isoforms vs. control cells. Data expressed as average ± SE; ^*^p < 0.05, NS - not significant when compared to control (unpaired *t*-test). (**E**) DNA repair array showing the fold change in the expression of genes involved in the DNA-damage repair in Ovcar4-BRD4-S cell line relative to the control (Ovcar4 parental cells). Data expressed as average ± SE.

We observed that the overexpression of BRD4-S in Ovcar4 cell line significantly increased the number of cells arrested in G2/M phase and polyploid cells. Since the G2/M cell cycle arrest is often associated with accumulation of DNA defects in cells, we sought to investigate the presence of DNA damage in the context of BRD4 isoforms overexpression or knockdown by quantifying the number of cells with pH2AX foci (a DNA damage marker). Our results revealed that a genetic manipulation of the BRD4-S expression (overexpression or knockdown) in ovarian cancer cells resulted in a significantly higher number of pH2AX foci (Figure 4A–4C). In addition, BRD4-S-KD cells showed significantly higher number of mitotic cells with DNA damage (Figure 4B, star, and Figure 4D). In contrast, no significant difference was observed when we modified the expression of BRD4-L isoform (Figure 4A, 4B). These findings demonstrated that any significant changes in the expression of BRD4-S isoform contribute to increased DNA damage in ovarian carcinoma.

To get an insight into the potential mechanism by which the changes in expression levels of BRD4-S isoform promote DNA damage in ovarian carcinoma, we performed an mRNA expression array investigating genes involved in the DNA-damage repair process. In comparison to parental Ovcar4 cells, the Ovcar4-BRD4-S cells showed an increased expression of several genes involved in DNA-damage repair, most significantly *ATM*, *CCNO*, *BARD1*, *BRCA2* and *RAD9A* (Figure 4E). Specifically, the expression levels of *ATM* in Ovcar4-BRD4-S were approximately 4-times higher vs. control cells. ATM (ataxia telangiectasia mutated) is a kinase that is promptly activated in the presence of DNA damage caused by double strand breaks [35, 36] or stalled replication forks [37]. Under exposure to ionizing radiation, BRD4-S is known for altering the chromatin conformation shielding the DNA from ATM signaling pathway proteins [16, 33]. Interestingly, the second highest expressed gene involved in DNA damage and repair in BRD4-S overexpressing cells is cyclin O (*CCNO)*, which is a novel finding. Cyclin O has been recently identified as a key gene associated with chemotherapy resistance in HGSOC [38], however no connection between BRD4 isoforms and *CCNO* expression has been reported to date.

### Ovarian carcinoma cells overexpressing BRD4 isoforms are highly resistant to cisplatin *in vitro*

**Figure 5:**
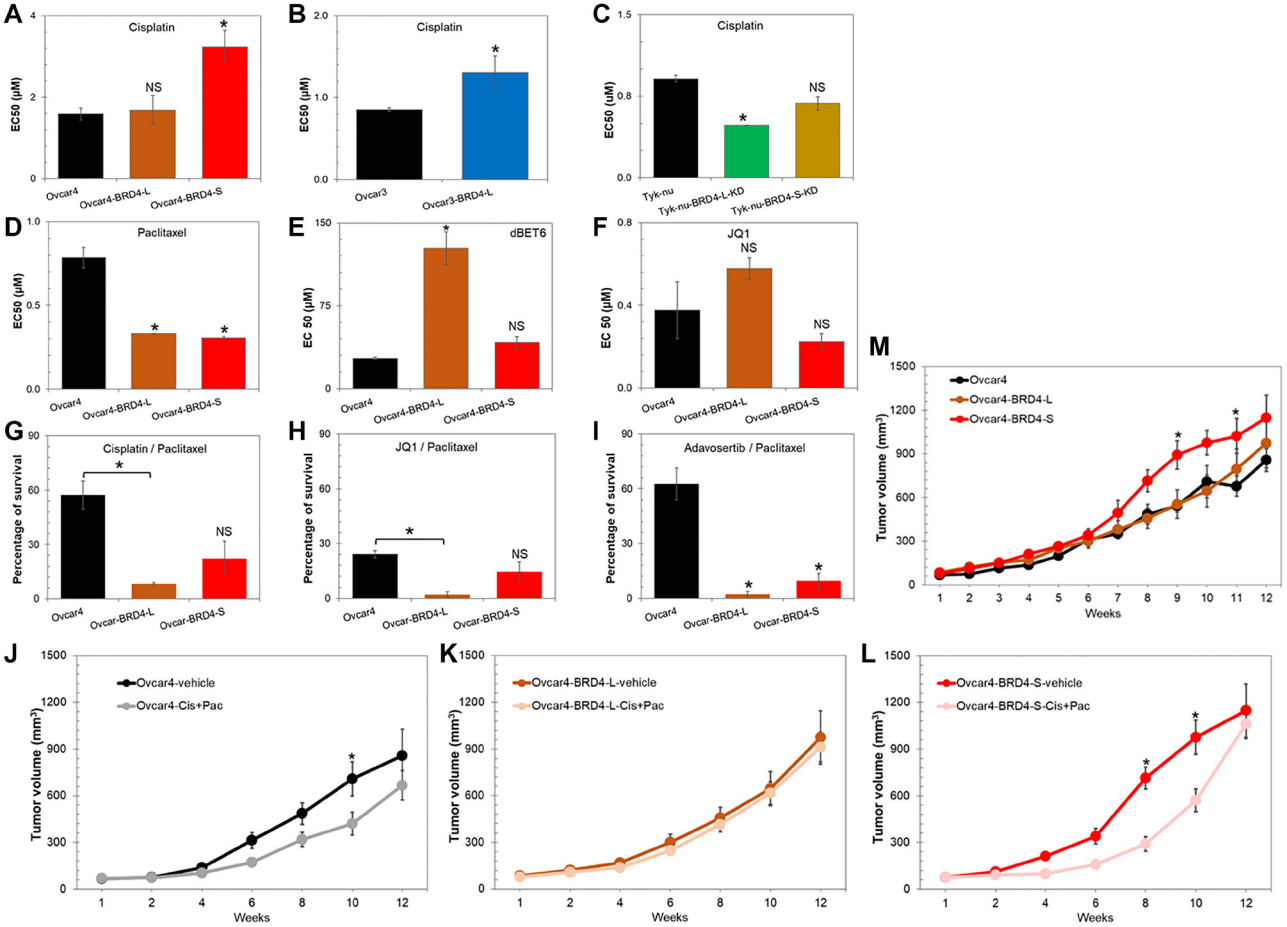
Overexpression of BRD4 isoforms in ovarian carcinoma promotes chemotherapy resistance *in vitro* and *in vivo*. (**A**–**I**) Drug dose-response assay performed in cell lines with overexpression or knockdown of respective BRD4 isoforms. Data are presented as EC50 values of selected compounds (cisplatin (A–C), paclitaxel (D), dBET6 (E), JQ1 (F), cisplatin/paclitaxel (G), JQ1/paclitaxel (H), and adavosertib/paclitaxel in I) assessed for each cell line. The lower the EC50 value, the more potent drug in killing cancer cells. Data expressed as average ± SE; ^*^p < 0.05, NS - not significant when compared to control (unpaired *t*-test). (**J**–**M**) Assessment of tumor growth rate *in vivo* of subcutaneously implanted Ovcar4 cell lines with and without overexpression of BRD4 isoforms in NOD/SCID mice. Animals received 3 weekly cycles of cisplatin/paclitaxel chemotherapy or vehicle control. Data are expressed as average ± SE; ^*^p < 0.05 (one-way ANOVA followed by Tukey’s post-hoc).

Following the discovery that Ovcar4 cells overexpressing BRD4-S isoform showed an extensive DNA damage, we inquired about their response to DNA-damaging agents, such as cisplatin, which is used as a standard care treatment for ovarian carcinoma patients. We performed drug dose-response assay to test survival of cells exposed to anti-cancer agents. We exposed selected cell lines to various concentrations of cisplatin and calculated the half-maximal effective concentration (EC50) of cisplatin for each cell line. Exposure of Ovcar4-BRD4-S cells to cisplatin *in vitro* (Figure 5A) revealed that these cells are significantly more resistant to cisplatin than their parental Ovcar4 cells. However, no difference in response to cisplatin was observed between Ovcar4-BRD4-L and control cells (Figure 5A). The analysis of other cell lines demonstrated that Ovcar3-BRD4-L cells are significantly more resistant to cisplatin vs. parental Ovcar3 cells (Figure 5B). In contrast, knockdown of BRD4 isoforms made Tyk-nu cell line significantly more sensitive to cisplatin (Figure 5C). Collectively, these results indicate that cells with higher levels of either BRD4-L or BRD4-S are substantially more resistant to cisplatin *in vitro*.

### Paclitaxel and adavosertib show potent antitumor activity in BRD4 overexpressing cancer cells *in vitro*

Next, we investigated the response of Ovcar4 cell lines to paclitaxel (standard-care chemotherapy) as well as their response to BRD4 inhibitors JQ1 and dBET6. Both, Ovcar4-BRD4-L and Ovcar4-BRD4-S were significantly more sensitive to paclitaxel than Ovcar4 cell line (Figure 5D), suggesting that paclitaxel alone, or in combination with other drugs, is likely an effective treatment for BRD4 overexpressing tumors. When we tested the efficacy of BRD4 inhibitors, we made an intriguing observation that the Ovcar4-BRD4-L cells were more resistant to these drugs than control cells lacking the BRD4 overexpression (Figure 5E, 5F). Specifically, Ovcar4-BRD4-L cells were significantly more resistant to the BRD4 degrader dBET6, or showed tendency to higher resistance to JQ1 agent when compared with control cells. Further, we found no difference in response to BRD4 inhibitors of cell overexpressing BRD4-S isoform vs. parental control cells (Figure 5E, 5F).

Since we found that Ovcar4 cells overexpressing BRD4 isoforms are particularly sensitive to paclitaxel, we then investigated if combination of paclitaxel and other anti-cancer compounds can enhance the antitumor effect even further (Figure 5G–5I). It is known that paclitaxel stabilizes microtubules, which disrupts mitosis. Exposure to paclitaxel results in the cell cycle arrest in G2/M phase, which is often followed by the activation of pro-apoptotic signaling pathways and cell death [39]. Thus, we hypothesized that the combination of paclitaxel with a drug that disrupts the same phase of the cell cycle (G2/M phase) could lead to an increased antitumor efficacy. We investigated the effect of a combination of paclitaxel with Wee-1 inhibitor adavosertib. Adavosertib abrogates the function of wee-1, which is a G2/M cell cycle checkpoint regulator that prevents cells with DNA-damage from entering mitosis. In addition, we tested the combination of paclitaxel with cisplatin or the bromodomain inhibitor JQ1. Overall, Ovcar4 cells overexpressing BRD4 isoforms were more sensitive to all the investigated combination therapies (Figure 5G–5I). The most significant antitumor response was observed in cell lines overexpressing either BRD4-L or BRD4-S that were simultaneously exposed to adavosertib and paclitaxel (Figure 5I).

### Overexpression of BRD4 isoforms in ovarian carcinoma promotes chemotherapy resistance *in vivo*

Next, we investigated if the ovarian cancer phenotype associated with the overexpression of individual BRD4 isoforms, such as increased cell proliferation or chemoresistance could be recapitulated *in vivo*. We implanted subcutaneously into NOD/SCID mice Ovcar4 cells with and without the overexpression of BRD4-L or BRD4-S, and assessed tumor growth rate *in vivo*. Results revealed that Ovcar4-BRD4-S tumor grew faster than Ovcar4 or Ovcar4-BRD4-L tumors (Figure 5M), which reflects the more aggressive phenotype of Ovcar4-BRD4-S cells observed *in vitro* (Figure 2A). Further, we noted that the BRD4-L overexpression did not significantly affect the Ovcar4 tumor growth rate, while *in vitro* these cells tended to proliferate slower when compared with controls (Figure 5M).

Next, we investigated the response of Ovcar4 tumor models overexpressing BRD4 isoforms *in vivo* to a combination therapy of cisplatin and paclitaxel (first-line treatment for ovarian carcinoma patients). Results revealed that parental Ovcar4 tumors showed some degree of resistance to cisplatin/paclitaxel therapy reflected as a slower tumor growth rate (Figure 5J). The BRD4-L overexpressing tumors demonstrated a complete lack of response to chemotherapy, the chemotherapy-treated tumors grew at same rate as those treated with vehicle control (Figure 5K). In contrast, the BRD4-S overexpressing tumors showed an initial response to cisplatin/paclitaxel treatment, however after treatment cessation, the tumors accelerated their growth reaching the same size as control tumors at endpoint of the experiment (Figure 5L). Taken together, our *in vivo* findings indicate that the overexpression of BRD4, particularly BRD4-L isoform is associated with chemotherapy resistance in ovarian cancer.

### Distinct correlation between BRD4 isoform splicing variants and BRD4 protein expression in ovarian PDX models

**Figure 6:**
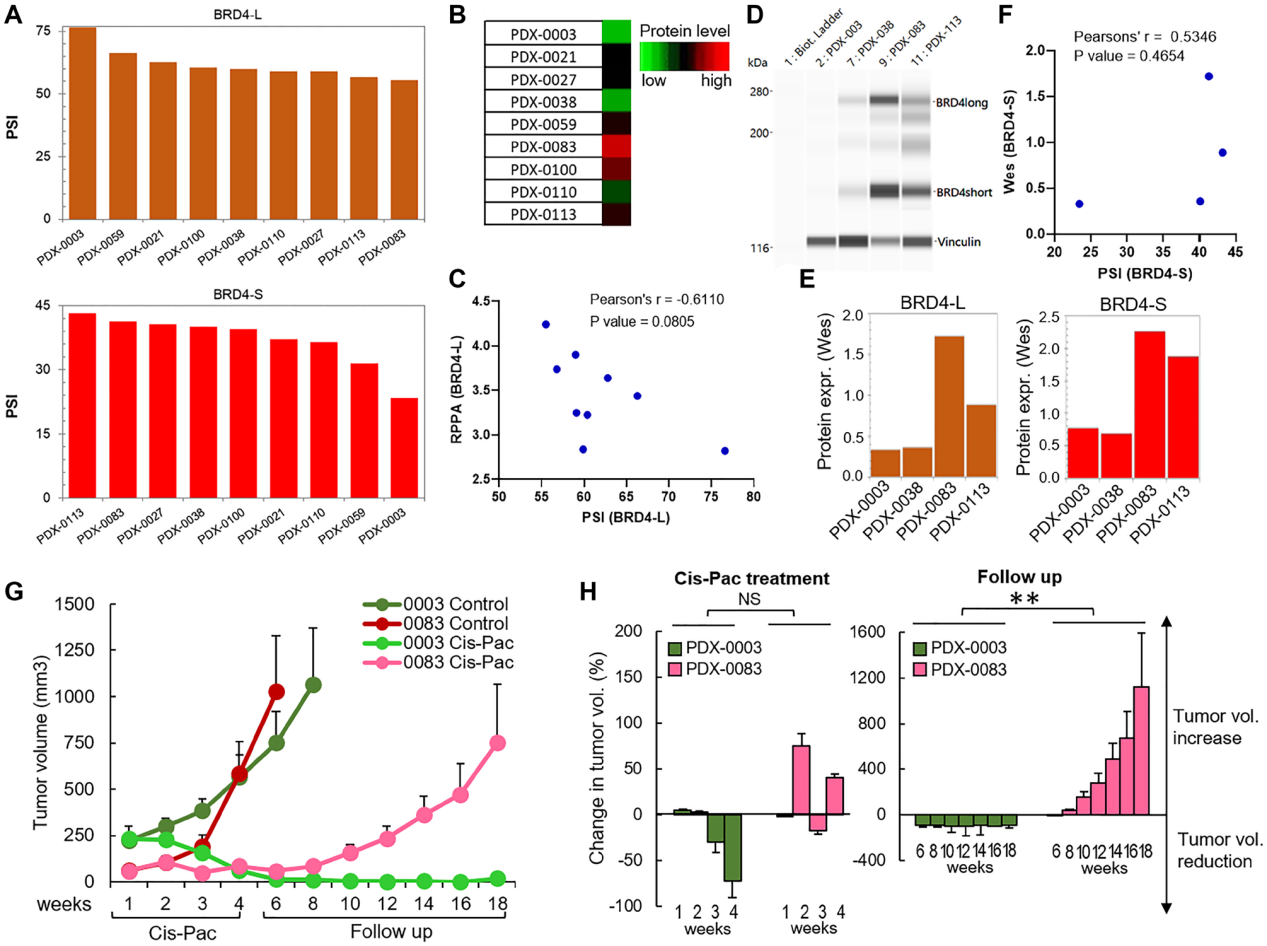
Patient-derived xenografts with high expression of BRD4-L and BRD4-S are resistant to cisplatin/paclitaxel chemotherapy. (**A**) RNA-Seq data were used to calculate the percentage of spliced-in (PSI) index of BRD4-L mRNA and BRD4-S mRNA. PSI represents the percentage of specific BRD4 isoform transcripts out of all BRD4 isoforms transcripts. (**B**) RPPA analysis (presented as a heatmap) illustrates quantification of BRD4-L protein expression in ovarian PDXs. PDXs with the highest BRD4-L expression are denoted in red, while those with the lowest BRD4-L expression are denoted in green. (**C**) The correlation between BRD4-L isoform transcript and protein expression was assessed by the Pearson’s correlation analysis, which revealed a negative correlation between mRNA and protein levels. (**D**) Wes image of selected PDX models showed a similar BRD4-L protein expression profile (quantified in **E**) as observed in RPPA results. Vinculin was used as a loading control. (**F**) The Pearson’s correlation analysis revealed a positive correlation between BRD4-S mRNA and protein levels. (**G**) Assessment of tumor growth rate *in vivo* and response to cisplatin/paclitaxel chemotherapy of subcutaneously implanted BRD4-high (PDX-0083) and BRD4-low (PDX-0003) tumor models. Animals received 4 weekly cycles of cisplatin/paclitaxel chemotherapy or vehicle control. (**H**) Waterfall plots show the response of individual PDXs to cisplatin/paclitaxel treatment during therapy (week 1–4) and during follow up period (week 6–18). Percentage change of tumor volume values below 0 indicate tumor regression, while the values above 0 indicate increased tumor volume when compared to initial tumor volume (prior treatment). Data expressed as average ± SE; ^**^p < 0.01, NS - not significant when compared to control (unpaired *t*-test).

Using our collection of patient-derived xenografts (PDXs) representing high-grade serous ovarian cancer (Supplementary Table 1 [40]), we analyzed the correlation between the expression of BRD4 isoforms transcript vs. protein. First, we performed RNA-Seq analysis of PDX samples followed by transcript quantification of BRD4 isoforms (Supplementary Table 2). Then, the transcript counts were used to calculate the percentage of spliced-in (PSI) index [41] of BRD4-L and BRD4-S (Figure 6A and Supplementary Table 2). The PSI numbers were used to estimate the splicing ratio of BRD4-L and BRD4-S isoforms (Supplementary Table 2). PSI and splicing ratio values revealed that PDX-0083 and PDX-0113 had the highest expression of BRD4-S isoform and, in consequence, the lowest BRD4-L to BRD4-S splicing ratio. Additionally, we identified PDX-0003 as having the highest expression of BRD4-L transcript and the lowest levels of BRD4-S transcript resulting in a high BRD4-L/BRD4-S ratio of 3.3.

Further, to test if BRD4 transcript levels correlate with BRD4 protein expression, we quantified BRD4 proteins by Reverse Phase Protein Array (RPPA) using the same PDX models. We quantified only BRD4-L isoform by RPPA, since the BRD4-L-specific antibody was the only validated BRD4 antibody for RPPA method. RPPA results showed that PDX-0083 had the highest protein expression of BRD4-L, while PDX-0003 demonstrated the lowest BRD4-L levels (Figure 6B), which we confirmed by Wes (ProteinSimple) analysis (Figure 6D). However, we have not observed a positive correlation between PSI values of BRD4-L splice variant and BRD4-L protein expression (Figure 6A–6C). In contrary, higher PSI values of BRD4-L tended to correlate with lower protein levels of BRD4-L (without reaching statistical significance), which indicates a potential post-translational regulation leading to a BRD4-L protein instability (Figure 6C). In contrast, the BRD4-S PSI values showed a noticeable positive correlation with BRD4-S protein levels (Figure 6D–6F).

### Patient-derived xenografts with high expression of BRD4-L and BRD4-S are resistant to cisplatin/paclitaxel chemotherapy

Next, we used PDX models to evaluate an impact of BRD4 isoform overexpression on treatment response to cisplatin/paclitaxel combination therapy *in vivo*. We selected PDX-0083, which showed the highest levels of BRD4-L and BRD4-S, and PDX-0003, which is characterized by the lowest expression of those isoforms. The treatment consisted of weekly injections of cisplatin and paclitaxel. Tumor measurements were recorded weekly during 4 cycles of treatment. Results demonstrated that control PDX-0083 tumors (BRD4-high model) grew faster than control PDX-0003 tumors (BRD4-low model, Figure 6D, 6G). PDX-0083 reached an average tumor size of ~1,000 mm^3^ within 6 weeks since treatment initiation, while PDX-0003 reached the same tumor size after 8 weeks (Figure 6G). To evaluate the *in vivo* response to cisplatin/paclitaxel treatment of BRD4-high and BRD4-low PDX models, we adopted a previously described method for treatment response classification by Gao et al. group [42]. The treatment response was determined by comparing tumor volume change at time t to its baseline: % tumor volume change = ΔVolt = 100% × ((Vt – Vinitial) / Vinitial). The criteria for response were defined as follows: ΔVolt < −40% - complete response (CR); ΔVolt < −20% - partial response (PR); ΔVolt < 30% - stable disease (SD); ΔVolt > 30% - progressive disease (PD). Based on this classification, BRD4-low PDX-0003 showed complete response following cisplatin/paclitaxel treatment (at week 4). In contrast, the BRD4-high PDX-0083 showed partial response at week 2, however at week 4, PDX-003 treatment response transitioned to progressive disease (Figure 6H). Next, in follow up period after cessation of chemotherapy, we observed profound differences in tumor recurrence status between BRD4-high and BRD4-low PDX modes. The BRD4-low PDX-0003 remained in complete response for at least 18 weeks, while BRD4-high PDX-0083 promptly recurred following treatment and showed progressive diseases status throughout follow up period (Figure 6F–6H).

## DISCUSSION

The amplification of *BRD4* gene is frequently associated with chemoresistance in HGSOC [8–10, 27], accompanied by considerable worse patient prognosis. Nonetheless, very little is known about the overexpression effects of BRD4 isoforms, BRD4 long (BRD4-L) and BRD4 short (BRD4-S), in this malignancy. In this study, for the first time, we investigated the BRD4 isoform expression patterns on a transcript and protein levels. Overall, our study shed a light on a complex regulation of BRD4 isoforms likely involving several regulatory mechanism (such as e.g., post-transcriptional and/or post-translation regulation), which resulted in a distinct correlation between transcript and protein expression of individual BRD4 isoforms. Our work has also led to several interesting findings. Results with the use of human ovarian cancer cell lines showed that the BRD4-L mRNA expression is required to generate both isoforms (BRD4-L and BRD4-S), and that the BRD4-S knockdown is associated with increased expression of BRD4-L. These findings highlights a potential compensatory mechanism where a reduction of BRD4-S expression triggers BRD4-L transcription, which is essential for generation of BRD4-S isoform. As another mechanism, by which BRD4-L could positively control BRD4-S transcription is its role in gene splicing regulation. It has been shown that BRD4 regulates splicing of multiple genes in normal and cancer cells [43–45] by interacting directly with the splicing machinery [45]. In the absence of BRD4, via knockdown or inhibition, there is a decrease of splicing of BRD4-regulated genes, and a consequent alteration of splicing patterns. The above studies have not specifically investigated the role of individual BRD4 isoforms in splicing process, however the analysis of reported data indicates that the BRD4-L isoform has been a major gene splicing regulator [43–45]. Based on our own and published data, it is likely that the BRD4-L regulates the splicing of BRD4-S transcript in ovarian cancer. However, further in-depth mechanistic studies are needed to support this hypothesis.

To improve clinical relevance of our studies investigating an impact of BRD4 isoforms abundance on ovarian cancer pathogenesis, we used PDX models derived from patients diagnosed with a high-grade serous ovarian carcinoma (HGSOC). PDXs have been shown to capture cellular and molecular characteristics of human tumors better than simpler cell line-based models and have been considered a valuable tool in preclinical research [46, 47]. In current study, we selected several PDX models from our published collection of well-established and extensively characterized HGSOC PDXs [40], and performed RNA-Seq and proteomic analysis to get insight into BRD4 isoforms abundance and splicing ratio. The RNA-Seq results revealed a range of BRD4 isoforms expression (BRD4-L: 55.5 – 76.6 PSI; BRD4-S: 23.4 – 43.2 PSI), and BRD4-L/BRD4-S splicing ratios (1.3 – 3.3, as assessed by spliced-in (PSI) index values [41]). Our data revealed that the BRD4-L mRNA was ~2 times more abundant than BRD4-S mRNA, which is in agreement with Wu SY et al. findings, where the authors reported 2:1 transcript ratio of BRD4-L to BRD4-S in breast cancer [21]. Interestingly, when analyzing the relationship between transcript and protein expression of BRD4-L isoform in ovarian PDXs, we observed that increased BRD4-L transcript levels tend to correlate with lower protein levels of BRD4-L. These findings indicates a potential post-translational regulation of BRD4-L, which for instance could lead to a BRD4-L protein instability. In fact, several mechanisms of post-translational modifications have been reported for BRD4 proteins, particularly ubiquitination and phosphorylation [48]. The latter is related to the biological functions of BRD4, such as transcriptional regulation, cofactor recruitment, and chromatin binding [48]. The ubiquitin-regulated degradation of BRD4 would be a particularly interesting subject of study in ovarian carcinoma. It is unknown if individual BRD4 isoforms protein stability is regulated via the same post-translational mechanism or a different mechanism leading to variable ratio of BRD4-L to BRD4-S as observed in our study. Studies show that in addition to *BRD4* gene amplification resulting in increased expression of BRD4 isoforms in ovarian cancer, the increased BRD4 protein abundance could be associated with decreased ubiquitination, or increased de-ubiquitination as observed in other types of tumors [48–51]. In contrast, when analyzing BRD4-S data in the same PDX lines, we observed a positive correlation between transcript and protein expression, where increased levels of mRNA corresponded to higher protein expression of BRD4-S isoform. Collectively, our findings revealed that while the BRD4-S transcript abundance reflects well the BRD4-S protein expression in ovarian tumors, the quantification of BRD4-L transcript abundance is not reliable method for predicting the BRD4-L protein expression. These factors should be taken into consideration when evaluating clinical tumor samples for BRD4 abundance. We recognize that a limitation of our work is a lack of in-depth understanding of transcriptional and post-transcriptional regulation of BRD4 isoforms. However, this work has been an important step towards our ongoing and future comprehensive studies providing a better insight into BRD4 isoforms genomic and proteomic regulation and oncogenic activity in ovarian cancer.

Our phenotypic data demonstrated that the increased expression of BRD4-S contributes to more aggressive tumor phenotype reflected as faster cell proliferation and improved cell survival in anchorage-independent conditions *in vitro*, as well as faster tumor growth *in vivo*. Our findings are in agreement with previous studies performed with the use of breast cancer models [21, 23, 25], and transformed ovarian cells [7]. Our data also confirmed what has been previously reported for breast cancer cells [18, 21] that BRD4-S and BRD4-L exhibit opposing roles in regulating cell proliferation *in vitro*, where BRD4-S promotes cell proliferation, while BRD4-L suppresses it. Despite distinct effects of the overexpression of individual BRD4 isoforms on cell proliferation and tumor growth, both isoforms contributed to chemotherapy resistance in our study. We showed that the overexpression of either BRD4-L or BRD4-S in Ovcar4 cell line resulted in resistance of those cells to cisplatin *in vitro* and cisplatin/paclitaxel combination therapy *in vivo*. This observation was confirmed by BRD4 knockdown in Tyk-nu cells, which led to less aggressive tumor phenotype and increased sensitivity to chemotherapy.

Our *in vitro* data using human ovarian cancer cell lines revealed that the overexpression of BRD4-L or BRD4-S isoform contributes to resistance to platinum drugs such as cisplatin. Further *in vivo* data showed that the Ovcar4 cell line-based tumor model is highly resistant to cisplatin/paclitaxel treatment even prior overexpression of BRD4 isoforms, which is in agreement with literature [52]. We demonstrated that the overexpression of BRD4, particularly BRD4-L isoform in already chemoresistant Ovcar4 cell line augmented chemoresistant phenotype even more. We observed a complete lack of response to chemotherapy *in vivo* in Ovcar4-BRD4-L tumors, and an initial treatment response followed by a rapid tumor recurrence in Ovcar4-BRD4-S tumors. To validate these finding with a more clinically relevant tumor models, we performed *in vivo* experiments using chemotherapy-naïve patient-derived models of ovarian cancer exposed to cisplatin/paclitaxel chemotherapy. *In vivo* studies using BRD4-low PDX model showed a complete and durable response to cisplatin/paclitaxel regimen during treatment and during follow up period. In contrast, BRD4-high model demonstrated a partial response to cisplatin/paclitaxel treatment, which was followed by a rapid tumor recurrence after therapy cessation. Our findings are in agreement with a previously described role of BRD4 in promoting chemoresistance via mechanisms such as regulation of ALDH activity [29] and promotion of DNA damage repair [32]. However, since our work have been performed with a small number of cell line-based or PDX tumor models, this suggests the need for further validation by a comprehensive study including larger number of clinically relevant ovarian cancer models. Our data also revealed a strong positive correlation between BRD4 overexpression status and chemoresistance in ovarian cancer, which could serve as a prognostic tool to predict chemotherapy treatment outcomes in ovarian cancer patients.

Our studies revealed that the overexpression of BRD4-S promotes substantial DNA damage in cells, which correlates with the cell cycle arrest and formation of polyploid giant cells. Floyd et al., demonstrated that the BRD4-S shields the chromatin from DDR proteins, particularly ATM, under the exposure to ionizing radiation [16], however the authors have not detected DNA damage in cells overexpressing BRD4-S under normal conditions (prior ionizing radiation). In contrast, we observed high levels of DNA-damage in Ovcar4-BRD4-S cells in normal conditions, which was associated with a substantial increase of ATM expression. As mentioned previously, ATM is activated in the presence of DNA damage caused by double strand breaks [35, 36] or stalled replication forks [37]. Furthermore, the increased DNA damage in Ovcar4-BRD4-S cells is likely the mechanism promoting cell cycle arrest in G2/M-phase, and the formation of polyploid giant cells. The formation of polyploid cells and G2/M arrest are co-occurring events, often observed in ovarian carcinoma cells [53, 54]. Polyploid cells are formed when cancer cells undergo endoreplication following DNA damage that leads to severe mitotic stress [55]. These cells can reach a quiescent state, followed by periods of asymmetric mitotic divisions similarly as chemoresistant cells [54]. It has been previously proposed that polyploid giant cells function similarly as cancer stem-like cells contributing to development of chemoresistance [34, 53, 54]. Collectively, we propose that one of the mechanisms driving chemoresistance in ovarian carcinoma with amplified *BRD4* could be an increased DNA damage and generation of polyploid giant cells that arise as a result of BRD4-S overexpression. Interestingly, we also noticed that Tyk-nu cells depleted of BRD4-S showed a high level of DNA damage. These findings suggest that an optimal expression of BRD4-S in cells is required to spatiotemporally coordinate transcription [18, 56]. For instance, it has been reported that the absence of BRD4 leads to an accumulation of RNA:DNA hybrids, called R-loops [56], which collide with the replication machinery causing replication stress and consequent DNA damage, similarly as we observed in Tyk-nu-BRD4-S-KD in our study. Moreover, the presence of DNA-damage in cells with overexpression or knockdown of BRD4-S reinforces the importance of maintaining appropriate levels of BRD4-S expression for proper function of the DNA-damage repair machinery.

Our further studies seeking alternative therapies to effectively target BRD4 overexpressing tumors identified a promising combination therapy consisting of paclitaxel and the Wee-1 inhibitor adavosertib. In response to DNA damage, Wee-1 induces cell cycle arrest during G2/M checkpoint, by promoting CDK1 inhibitory phosphorylation [57]. Adavosertib inhibits Wee-1 pathway [58], which forces cancer cells to prematurely enter mitosis, while also impairing DNA damage repair, and consequently promoting cell death [59, 60]. Since Ovcar4-BRD4-S cells show severe DNA damage and are largely arrested in G2/M phase, thus our data indicate that these cells could be particularly vulnerable to Wee-1 inhibitor adavosertib. Furthermore, an overexpression of Ovcar4-BRD4-S leads to a generation of chemoresistant polyploid giant cells with cancer stem-like cell features, making this cell line a relevant model to study alternative treatment options such as Wee-1 inhibitors. In our *in vitro* studies, we observed that a treatment of cells with paclitaxel/adavosertib was very effective in killing Ovcar4-BRD4-L and Ovcar4-BRD4-S cells. Our findings are consistent with previous studies showing that the adavosertib effectively inhibits ovarian cancer growth as a single agent in preclinical studies [61], and improves disease outcome of platinum-resistant ovarian carcinoma patients (evaluated in phase II clinical trials) [62–64].

Overall, our work revealed a strong positive correlation between BRD4 overexpression status and chemoresistance in ovarian cancer, which could be explored to develop a prognostic strategy to predict a patient response to platinum/taxane-based chemotherapy. However, since we showed that BRD4 transcript levels (especially BRD4-L isoform) does not reflect well the BRD4 protein levels in tumors, thus the BRD4 overexpression status in tumor tissues should be evaluated using immunohistochemistry or proteomic methods. Further, our drug screening experiments identified an adavosertib/paclitaxel combination therapy, which represents a strong candidate as alternative therapy for HGSOC patients with *BRD4* amplification offering hope for better treatment options for this devastating disease.

## MATERIALS AND METHODS

### Cell lines

All human ovarian cancer cell lines used in this work are commercial. Ovcar4 (NCI-DTP, cat. #OVCAR-4) and Ovcar3 (ATCC, cat. #HTB-161) cell lines were grown in RPMI media (Gibco, cat. #11875) with 5% fetal bovine serum (FBS, Biowest LLC, cat. #S1620). Tyk-nu cell lines (JCRB, cat. #JCRB0234.0) were grown in MEM media with GlutaMAX-I (Gibco, cat. #41090) with 5% FBS. Cells were cultured in a 5% CO_2_ tissue culture incubator at 37ºC. To ensure quality of data and to avoid issues associated with cell line misidentification, contamination or genetic drift, the cell lines were purchased from validated reliable source and cryopreserved in the lab cell line bank at low passage (passage 1–3). In addition, the Ovcar4 cell line that was obtained from our collaborator was authenticated by the ATCC, via ATCC’s Human short tandem repeat (STR) testing (cell authentication service).

### Animals

All animal procedures were approved by the OMRF’s Institutional Animal Care and Use Committee. At the endpoint, animals were humanely euthanized by CO_2_ inhalation as described in the approved IACUC animal use protocol (#22-01). Adult female, NOD/SCID mice (NOD.Cg-Prkdc^scid^/J; The Jackson Laboratory; Strain #001303) were used for *in vivo* drug response studies and evaluation of tumor growth rate. Adult female, NRG mice (NOD.Cg-Rag1^tm1Mom^ Il2rg^tm1Wjl^/SzJ; The Jackson Laboratory, Strain #007799) were used for PDX tumor growth rate and drug-response evaluation *in vivo*.

### Patient-derived xenografts

Patient-derived xenografts (PDX) were provided by the Patient-Derived Xenograft and Preclinical Therapeutics (PDX-PCT) Core at OMRF. All human tissues were processed in compliance with NIH regulations and institutional guidelines, approved by the Institutional Review Board at the OMRF (#15-14-OMRF) and at the University of Oklahoma (IRB #5286). The study was conducted in accordance with the tenets of the Declaration of Helsinki. Chemotherapy-naive tumors from ovarian cancer patients were obtained via core needle biopsy or surgical resection, following informed consent. Patients were adult women diagnosed and treated at the Stephenson Cancer Center at the University of Oklahoma. Tissue samples and clinical-pathological data collected by the PDX-PCT Core were de-identified at the time of collection. Detailed description of development and characterization of PDX models is provided in a previous publication [40].

### Overexpression and knockdown of BRD4 isoforms

The BRD4-L and BRD4-S DNA sequences were amplified via polymerase chain reaction (PCR) and cloned into LentiV_Blast (Addgene, #111887; [65]) overexpression plasmid, with the use of Cold Fusion cloning kit (System Biosciences, cat. #MC010A-1). The BRD4-L encoding sequence contains a high level of G-C bases, which results in a formation of DNA secondary structures. Thus, the amplification and sequencing of BRD4-L isoform was very challenging (as we described in a previous publication [66]). In order to amplify BRD4-L via PCR, we incubated the PCR template overnight with 0.5 M betaine (Sigma-Aldrich, cat. #B0300-5VL) prior to conducting PCR amplification. Betaine was also added to the PCR reaction at 0.5 M final concentration. Finally, 0.1 M betaine was added to all sequencing steps throughout the cloning process. In contrast, betaine was not used for BRD4-S amplification or sequencing. shRNAs for both isoforms were cloned into pLKO.1-blast plasmid (Addgene, #26655; [67]) with the use of T4 DNA ligase (New England Biolabs, cat. #M0202L). The PCR primers for BRD4 isoforms amplification and shRNA oligos can be found in the Supplementary Table 3). Molecular cloning was performed using High Efficiency 5-alpha Competent E.coli (NEB, cat. #C2987H) and clone selection was performed using 100 μg/mL of Ampicilin antibiotic (VWR, cat. #71003-352). Plasmids were purified with the CompactPrep Plasmid Maxi Kit (Qiagen, cat. #12863) in accordance with manufacturer protocol. Plasmids were verified via Sanger DNA sequencing, conducted at The OMRF Sanger DNA Sequencing facility. Lentivirus was generated by transfection of HEK293T cells (ATCC, cat. #CRL-3216) with engineered plasmids (LentiV_Blast-BRD4-L [66], LentiV_Blast-BRD4-S, pLKO.1-blast-shBRD4-L-5294 or pLKO.1-blast-shBRD4-S-9782) and lentivirus packaging and envelope plasmids (Addgene, psPAX2, cat. #12260 and pCMV-VSV-G, cat. #8454; [68]). Human ovarian cancer cell lines were infected with the respective lentivirus for 72 hours and selected with Basticidin (Gibco, cat. #A1113903) for 2 weeks to obtain BRD4 isoform-specific overexpression or knockdown cell lines.

### Colony formation assay

Colony formation assay (also known as a soft agar colony formation assay) was performed using soft agar in accordance with previously described protocol (Borowicz et al., 2014). Briefly, cells were cultured in 6-well plates in a mixture of 0.3% Difco™ Noble Agar (BD Biosciences, cat. #214220) and their respective media, which was added on a top of 0.5% noble agar layer. Next, cells were cultured for 11 days and then stained overnight with 200 μL of nitroblue tetrazolium chloride (VWR, cat. #VWR0329). Colonies were photographed with microscope Leica M205 FCA, using the Leica application suite X. Colony number and size was estimated using Image J, version 1.52a.

### Wes (ProteinSimple)

Wes from ProteinSimple (San Jose, CA, USA) is an automated capillary-based immunoassay used to detect and quantify proteins, where results are presented as Wes images resembling a traditional Western Blot data. Whole cell lysates were obtained by lysing 1 × 10^6^ of cells in Buffer B (25 mM Tris-HCl, pH 7.5, 0.42 M NaCl, 1.5 mM MgCl_2_, 0.5 mM EDTA, 1 mM DTT, 25% sucrose, 1 mM Na_3_VO_4_, and 1 × protease inhibitor cocktail) on ice for 15 min, followed by centrifugal clearing at 4°C for 10 min at 10,000 rpm. Protein concentration of cell lysate was measured using BioRad Quick Start Bradford 1× Dye Reagent (cat. #5000205) according to manufacturer protocol. Cellular proteins (0.5 mg/mL) were separated using 12–230 kDa Separation Module (cat. #SM-W004) or 66–440 kDa Separation Module (cat. #SM-W008) and Anti-Rabbit Detection Module (cat. #DM-001) and visualized using the standard instrument protocol. A list of antibodies used for Wes analysis can be found in the Supplementary Table 4. Results were analyzed using Compass for SW software, version 4.0.0.

### Reverse-transcriptase quantitative PCR (RT-qPCR)

An average of 3–4 × 10^6^ cells grown in exponential phase, were used for RNA extraction using the Qiagen RNeasy Minikit (cat. #74104). RNA was converted into cDNA using iScript™ supermix (Biorad, cat. #1708840) according to manufacturer’s protocol. Forward and reverse primers (500nM each) were mixed with 2× PowerUP™ SYBR™ Green Master Mix (Thermo Fisher Scientific, cat. #A25742), cDNA, and distilled water, to a total volume of 10 μl per reaction. A list of primers used for qPCR analysis can be found in the Supplementary Table 3. The qPCR reactions were performed using a Roche LightCycler^®^ 96. Standard curves were obtained for each primer set. All primers sets used were considered ideal for qPCR with efficiency of approximately 2 and standard curve slopes of approximately −3.3 Cq values obtained with the use of 100 ng of cDNA. A total of four replicates were performed per sample, per primer. Relative quantification was calculated by normalizing Cq values of each sample replicate by the average Cq values of actin B (dCq). Relative quantification to control sample was then calculated by normalizing each sample dCq values by the average dCq values of the control sample, per primer (ddCq). The final values were calculated using the formula E^-(ddCq)^, where E is the efficiency of each primer set, as calculated by the standard curve. Data is represented as average ± SE. Statistical significant differences between samples were reached when the relative gene expression was higher than 2 fold or lower than 0.5-fold change.

### Human DNA repair mechanism array

An average of 3–4 × 10^6^ cells were used for RNA extraction using the Qiagen RNeasy Minikit (cat. #74104). RNA was converted into cDNA using iScript™ supermix (Biorad, cat. #1708840) according to manufacturer’s protocol. TaqMan^®^ Human DNA Repair Mechanism Array plates were obtained from Applied Biosystems (Thermo Fisher Scientific, cat. #4418773). cDNA was mixed with TaqMan^®^ Fast Advanced Master Mix (Thermo Fisher Scientific, cat. #4444557) prior to plate assembly in accordance with a provided protocol. The same concentration of cDNA was used for all samples, and experiments were performed in independent biological duplicates. The qPCR reactions were performed using a Roche LightCycler^®^ 96. Relative quantification was assessed as described for RT-qPCR experiments. Data were represented as average ± SE.

### *In vitro* drug screening and drug dose-response assay

Cells were seeded on 96 well plates with standard culture media at a density of 2–3 × 10^3^ cells per well and allowed to attach overnight. The next day, selected drugs were added to respective cells after dilution in respective drug vehicle and culture media. In this study, the following compounds were used: Adavosertib (cat. #S1525), (+)-JQ1 (cat. #S7110), dBET6 (cat. #S8762) from Selleck Chemicals (Houston, TX, USA); paclitaxel (NDC #45963-613-53) and cisplatin (NDC #47781-609-25) from OU Pharmacists Care Center (Oklahoma City, OK). For drug screening, 1 μM of each drug (as single agent or in combination) was used. For half-maximal effective concentration (EC50) calculation, 11 different drug doses were prepared by serial dilution ranging from 0.1 to 100 μM. After 96 hours of incubation of cells with respective drugs, cell survival assay was performed using BioVision “Quick proliferation assay kit” (cat. #K302-2500) according to manufacturer protocol. Experiments were performed in triplicates and two independent experiments were performed for each cell line/drug combination. Control cells were incubated for 96 hours in culture media without drug(s). EC50 values were calculated using Gen5 software, version 3.02.

### Selected reaction monitoring mass spectrometry

The quantification of protein abundance of BRD4 isoforms in ovarian cancer cell lines was performed using selected reaction monitoring (SRM) mass spectrometry. Whole cell lysates were obtained by lysing 1 × 10^6^ of cells in Buffer B (as described for Wes immunoassay) and samples were prepared as previously described [69]. Briefly, lysates containing 50 μg of total protein were dehydrated and reconstituted in Laemmli buffer (VWR, cat. #76346-436) at 1 ug/uL and provided for SRM mass spectrometry analysis. A short gel was run with 20 uL of sample, fixed, and stained with GelCode™ Blue (Thermo Scientific, cat. #24594). Proteins were purified and digested with 1 μg of trypsin. The peptides produced after purification and digestion were evaporated to dryness, and reconstituted in 1% acetic acid (Fisher Scientific, cat. #MAX00746) for analysis. Aliquots of 5 μl of digested samples were analyzed using the Thermo Scientific TSQ Quantiva mass spectrometer system. BRD4 peptides were identified as specific to BRD4 isoform long (C-terminal peptides, SSSDSFEQFR and AASVVQPQPLVVVK). Other detected peptides (N-terminal, VDVIAGSSK, DAQEFGADVR, LNLPDYYK and NSNPDEIEIDFETLKPSTLR) were non-specific to individual BRD4 isoforms and reflected the abundance of all BRD4 isoforms combined (also referred as “other BRD4 isoforms). Four replicates were analyzed for each cell line and total amount of protein was estimated as average of the results obtained for all four samples. Protein concentration was determined using multiple validated peptide markers to determine abundance of each protein. Relative protein abundance of each sample was determined by normalization to bovine serum albumin (BSA) used as a non-endogenous internal standard and to control sample (parental cell line). The data were processed using Skyline version 3.7.0.10940 [70].

### *In vivo* tumor growth monitoring and drug treatment regimen - cell line-based tumor models

Adult female NOD/SCID mice were injected subcutaneously (SQ) with 3 × 10^6^ of Ovcar4, Ovcar4-BRD4-L or Ovcar4-BRD4-S cells. Harvested cells were counted and suspended in 50% Matrigel in HBBS (from Corning, VWR, cat. #47743-720). Tumors were measured weekly, using a caliper, and the tumor volume was calculated using the formula (L × W^2^)/2. Drug treatment was initiated when the tumors reached approximately 50–100 mm^3^. Mice were randomized and assigned into respective treatment groups. Control group received IV injection of PBS (Gibco, cat. #MRGF-6230) twice a week, for 3 weeks. Cisplatin and Paclitaxel (Cis/Pac) group received IV injection of 5 mg/kg of Cisplatin (NDC #N47781-609-25) once a week and IV injection of 10 mg/kg of paclitaxel (NDC #45963-613-53) once a week, for 3 weeks. Animals were euthanized 12 weeks after treatment initiation. Data were expressed as average ± SE.

### *In vivo* tumor growth monitoring and drug treatment regimen - PDX tumor models

PDX-0083 and PDX-0003 were implanted subcutaneously into adult female NRG mice, in accordance to previously published protocol [40]. Briefly, frozen/thawed tumors were surgically implanted in the dorsal flank of mice (*N* = 5) and animals were monitored weekly for tumor growth. Drug treatment was initiated when the tumors reached 50 mm^3^ (PDX-0083) or 200 mm^3^ (PDX-0003). Mice were randomized, assigned into respective treatment groups, and treated with PBS (vehicle control) or Cis/Pac as described for NOD/SCID mice harboring Ovcar4 tumors. Control tumors were measured until reaching ~1,000 mm^3^ of size and Cis/Pac treated PDXs were monitored for 18 weeks. Data were expressed as average ± SE. In addition, to evaluate the *in vivo* response to Cis/Pac treatment of BRD4-high and BRD4-low PDX models, we adopted a previously described method for treatment response classification by Gao et al. group [42]. The treatment response was determined by comparing tumor volume change at time t to its baseline: % tumor volume change = ΔVolt = 100% × ((Vt – Vinitial)/Vinitial). The criteria for response were defined as follows: ΔVolt < −40% - complete response (CR); ΔVolt < −20% - partial response (PR); ΔVolt < 30% - stable disease (SD); ΔVolt > 30% - progressive disease (PD).

### RNA-Seq - PDX tumor models

Total RNA was extracted from PDX tumors using the Qiagen RNeasy Minikit (cat. #74104). Samples were processed for RNA-Seq analysis by the Clinical Genomics Center at OMRF. Prior to RNA-Seq, samples concentration and quality were confirmed. Sequencing libraries were generated using the Lexogen Quantseq library prep kit (cat. #015.96) according to manufacturer protocol. Libraries were checked for appropriate size and quantity and then pooled in equimolar amounts, as ascertained via fluorometric analyses. Final pools were quantified using qPCR on a Roche LightCycler 480 instrument with Kapa Biosystems Illumina Library Quantification reagents (cat. #KK4854). Sequencing was performed using custom primers on the Illumina Nextseq 500 instrument with High Output chemistry and 75bp single-ended reads. Raw Illumina output was uploaded into Galaxy web platform (http://www.usegalaxy.org/, [71]) and Salmon quant was used for transcript quantification analysis. Reads were quantified based on reference transcriptome Release 39 (GRCh38.p13), which was downloaded from the Gencode website (https://www.gencodegenes.org/human/). Reads were quantified in transcripts per million (TPM). Percentage of spliced-in (PSI) index was calculated by dividing TPM values of each isoform by total TPM values of all BRD4 isoforms. Splicing ratio was estimated by dividing the PSI of the BRD4-L by the PSI of the BRD4-S. The RNA-Seq data presented here are part of a larger unpublished data set. Thus, for the purpose of this study only the data representing BRD4 isoforms expression were included.

### Reverse Phase Protein Array (RPPA) - PDX tumor models

Reverse Phase Protein Array (RPPA) of PDX tumor models was used to evaluate the protein expression of BRD4-L (only BRD4-L specific antibody was available for RPPA assay). Samples were processed and analyzed by the Functional Proteomics RPPA Core Facility at The UT MD Anderson Cancer Center and can be identified by the set number “RPPA CORE 01072019_155”. Briefly, tissue lysates were probed with BRD4-L antibody (Cell Signaling Technology, cat. #13440) and visualized by 3, 3 -diaminobenzidine (DAB) colorimetric reaction. Slides were scanned on a Huron Tissue Scope scanner to produce 16-bit TIFF images. Sample spots in TIFF images were identified and quantified by array-Pro Analyzer. Relative protein levels for each sample were determined and designated as log2 values (RawLog2). Data points were then normalized for protein loading and designated normalized linear values (NormLinear). For clustering analysis, NormLinear values were transformed to log2 (NormLog2) and then the median-centered sample was defined with a value “0”. The heatmaps were generated by the UT MD Anderson Cancer Center Department of Bioinformatics and Computational Biology using Cluster 3.0 as a hierarchical cluster using Pearson Correlation and a center metric. The RPPA data presented here are part of a larger unpublished data set. Thus, for the purpose of this study only the data representing BRD4-L expression were included.

### 3T5 Cell proliferation assay

The 3T5 cell proliferation assay was performed by plating 5 × 10^5^ cells per 10 cm tissue culture plate (each cell line was set up in triplicate), followed by counting and re-plating at the same density every 3 days for 12 days. Population doubling time was calculated using the formula ln(post-3-day cell count/5 × 10^5^)/ln(2). The given population doubling time was added to the cumulative doubling time of the previous count. Data were presented as average ± SD.

### Mitotic index and pH2AX foci quantification

Mitotic index and pH2AX foci were quantified by platting 1 × 10^5^ cells onto Falcon^®^ 8-chamber culture slides (cat. #354118). Cells were allowed to attach overnight and were fixed in 4% paraformaldehyde solution (Electron Microscopy Sciences, cat. #100503-917). Standard immunofluorescence was carried out for α-tubulin (Cell Signaling, cat. #8058) and pH2AX (Cell Signaling, cat. #9718) using secondary anti-rabbit antibody Alexa Fluor 594 conjugate (Cell Signaling cat. #8889), and DAPI staining (Sigma, cat. #D9542) for DNA counterstaining. Slides were photographed using a Zeiss AxioObserver.Z1 fluorescence microscope and at least 5 different fields were captured for each biological replicate. A total of 4 independent biological replicates were photographed per each cell type analyzed. Using image J software, version 1.52a, a total number of cells per field was estimated based on the number of DAPI-positive nuclei. A total number of mitotic cells was estimated based on recognition of mitotic figure visualized by DAPI and α-tubulin staining. A total number of cells positive for pH2AX was estimated, when the number of foci was larger than 20 per nuclei. The percentage of cells in mitosis, cells positive for pH2AX and mitotic cells positive for pH2AX (Tyk-nu cells only) was estimated based on a total number of cells. Data were represented as average ± SE.

### Cell cycle analysis

For cell cycle analysis, at least 1 × 10^6^ cells were fixed with chilled 70% ethanol overnight. Prior to flow cytometry analysis, the cells were washed with PBS and buffer (2% FBS, 0.1 %NaN_3_), and stained with BD Biosciences PI/RNAse staining buffer (cat. #550825) according to manufacturer protocol. Analysis was performed by recording approximately 5,000 events in a FACSCelesta instrument using FACSDiva software. FlowJo software (version 10.8.0) was used for quantification of percentage of cells present in each cell cycle phase and number of polyploid cells. At least two independent experiments were performed for each cell line. Data were presented as average ± SE.

## SUPPLEMENTARY MATERIALS



## Abbreviations

HGSOC: High-Grade Serous Ovarian Carcinoma
TCGA: The Cancer Genome Atlas
BRD4: Bromodomain containing protein 4
BRD4-L: BRD4 long isoform
BRD4-S: BRD4 short isoform
DDR: DNA damage repair
PDXs: patient-derived xenografts
OC: ovarian carcinoma
shRNA: short hairpin RNAs
MI: mitotic index
ATM: ataxia telangiectasia mutated
CCNO: cyclin O
EC50: half-maximal effective concentration value
PSI: percentage of spliced-in index
RPPA: Reverse-Phase Protein Array
STR: short tandem repeat
PCR: polymerase chain reaction
SQ: subcutaneous
IV: intravenous injection
TPM: transcripts per million
