# Supplementary material for: Increased expression of BRD4 isoforms long (BRD4-L) and short (BRD4-S) promotes chemotherapy resistance in high-grade serous ovarian carcinoma
Source: Genes Cancer. 2023 Sep 12;14:56–76. doi: 10.18632/genesandcancer.233 (PMC10496930; doi:10.18632/genesandcancer.233)
Supplement: Supplementary file 1 [file ganc-14-233-s001.pdf]

Increased expression of BRD4 isoforms long (BRD4-L) and short (BRD4-S) promotes chemotherapy resistance in high-grade serous ovarian carcinoma

SUPPLEMENTARY MATERIALS

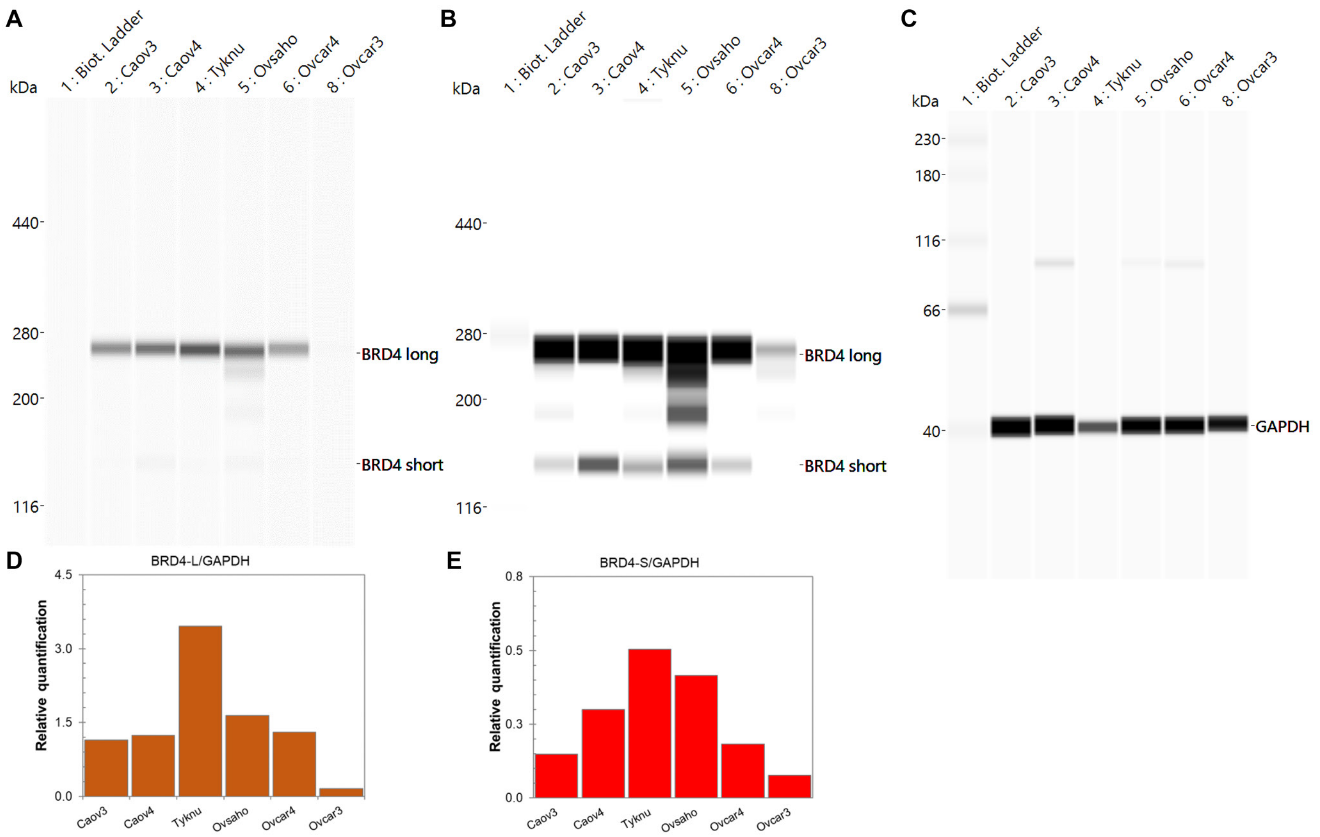

Supplementary Figure 1: Protein expression of BRD4 isoforms in ovarian carcinoma cell lines. (**A**–**C**) Wes (ProteinSimple) analysis of the expression of BRD4-L (A) and BRD4-S (B) vs. a control housekeeping protein GAPDH (C). (**D**, **E**) The relative quantification of protein expression of BRD4-L (D) and BRD4-S (E) normalized to GAPDH.

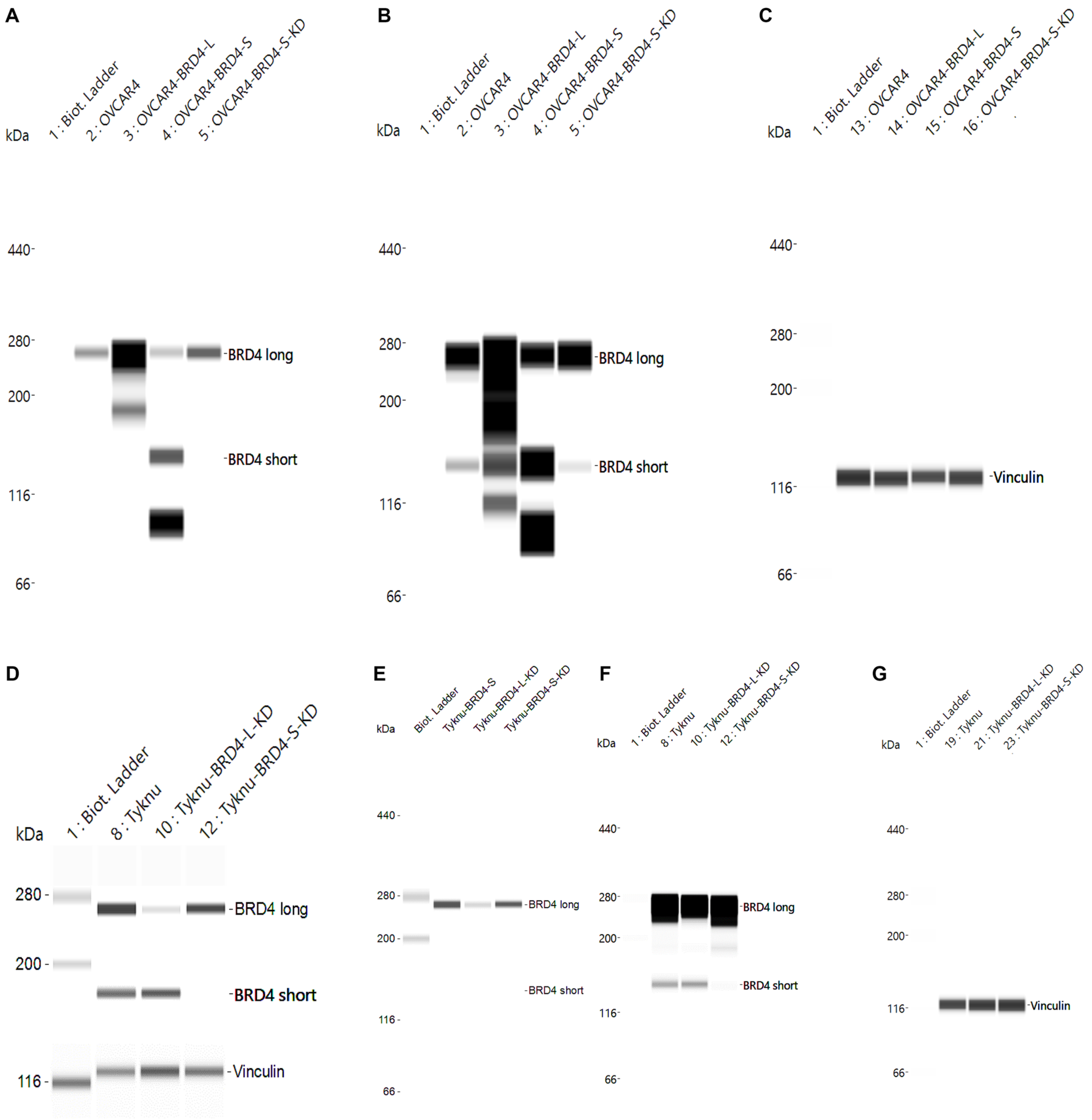

Supplementary Figure 2: Overexpression or knockdown of BRD4 isoforms in ovarian cancer cell lines validated by Wes (ProteinSimple). (**A**) Wes image showing BRD4-L and BRD4-S protein levels following their overexpression or knockdown in Ovcar4 cell line. (**B**) Higher exposure of the same Wes image as in (A) to allow for a better visualization of differences in BRD4-S protein expression. (**C**) Wes image showing vinculin expression, which was used as a loading control. (**D**) Representative Wes Image showing expression levels of BRD4-L or BRD4-S following overexpression or knockdown of respective BRD4 isoforms in Tyk-nu cell line. (**E**) Wes image showing BRD4-L and BRD4-S protein levels following their knockdown in Tyk-nu cell line (low exposure of Wes image). (**F**) Higher exposure of the same Wes image as in (E) to allow for a better visualization of BRD4-S isoform, which is significantly less abundant than BRD4-L isoform in Tyk-nu cells. (**G**) Wes image showing vinculin expression, which was used as a loading control.

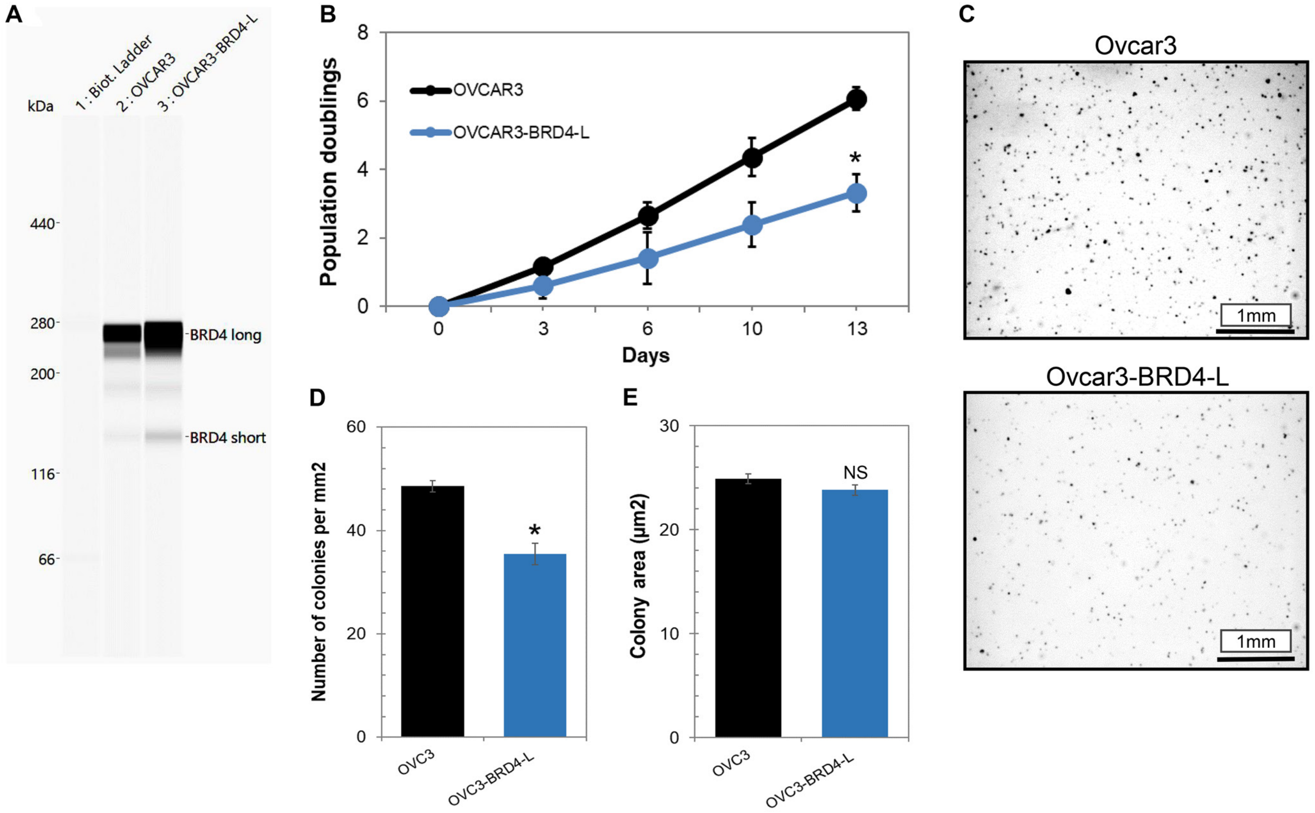

Supplementary Figure 3: Phenotype of Ovcar3 cell line following BRD4-L overexpression. (**A**) Wes image showing that the Ovcar3- BRD4-L cells expressed higher levels of BRD4 isoforms than the parental Ovcar3 cells. (**B**) Cumulative cell population doubling of Ovcar3 cell lines demonstrated that the Ovcar3-BRD4-L cells proliferated at a significantly slower rate than the parental cells. Data are expressed as average ± SD, (one-way ANOVA followed by Tukey's post-hoc, $^{*}p < 0.05$). (**C**–**E**) Soft agar colony formation assay evaluating the ability of Ovcar3 cells with and without BRD4-L overexpression to form colonies in anchorage-independent conditions. The number of colonies and their size were quantified in (D) and (E), respectively. The overexpression of BRD4-L in Ovcar3 cells results in generation of less colonies. Data are expressed as average ± SE, (unpaired *t*-test, $^{*}p < 0.05$).

A

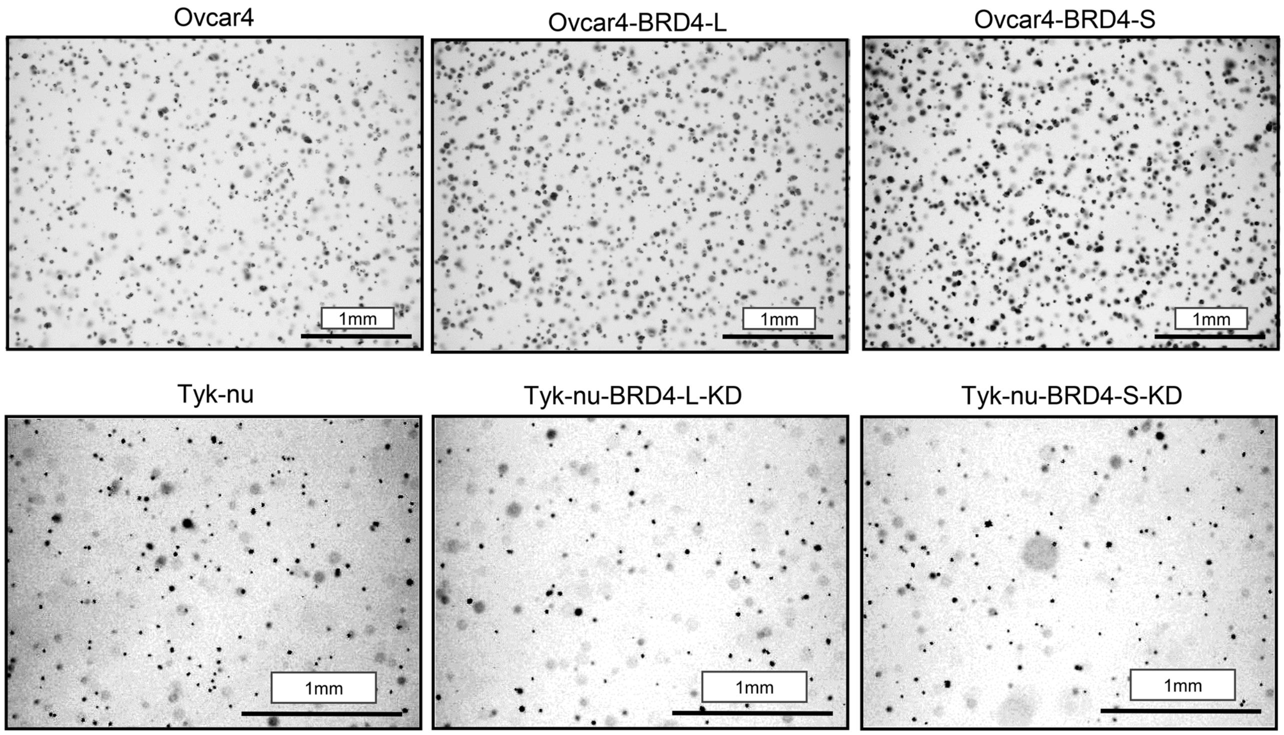

B Seeding density at Day 0:

Ovcar4 – 344 cells per mm^2
Ovcar4-BRD4-L – 367 cells per mm^2
Ovcar4-BRD4-S – 342 cells per mm^2

Tyk-nu – 587 cells per mm^2
Tyk-nu-BRD4-L-KD – 435 cells per mm^2
Tyk-nu-BRD4-S-KD – 390 cells per mm^2

Supplementary Figure 4: Anchorage-independent growth and colony formation of Ovcar4 and Tyk-nu cell lines following overexpression or knockdown of BRD4 isoforms. (**A**) Microscope images of respective cell lines suspended in soft agar medium at day 11 of the colony formation assay. Three images of each cell line were used to assess the number of colonies and their size. (**B**) Seeding density at day 0 of the colony formation assay, which was used to calculate the relative number of colonies per area at day 11 reported in the Figure 2.

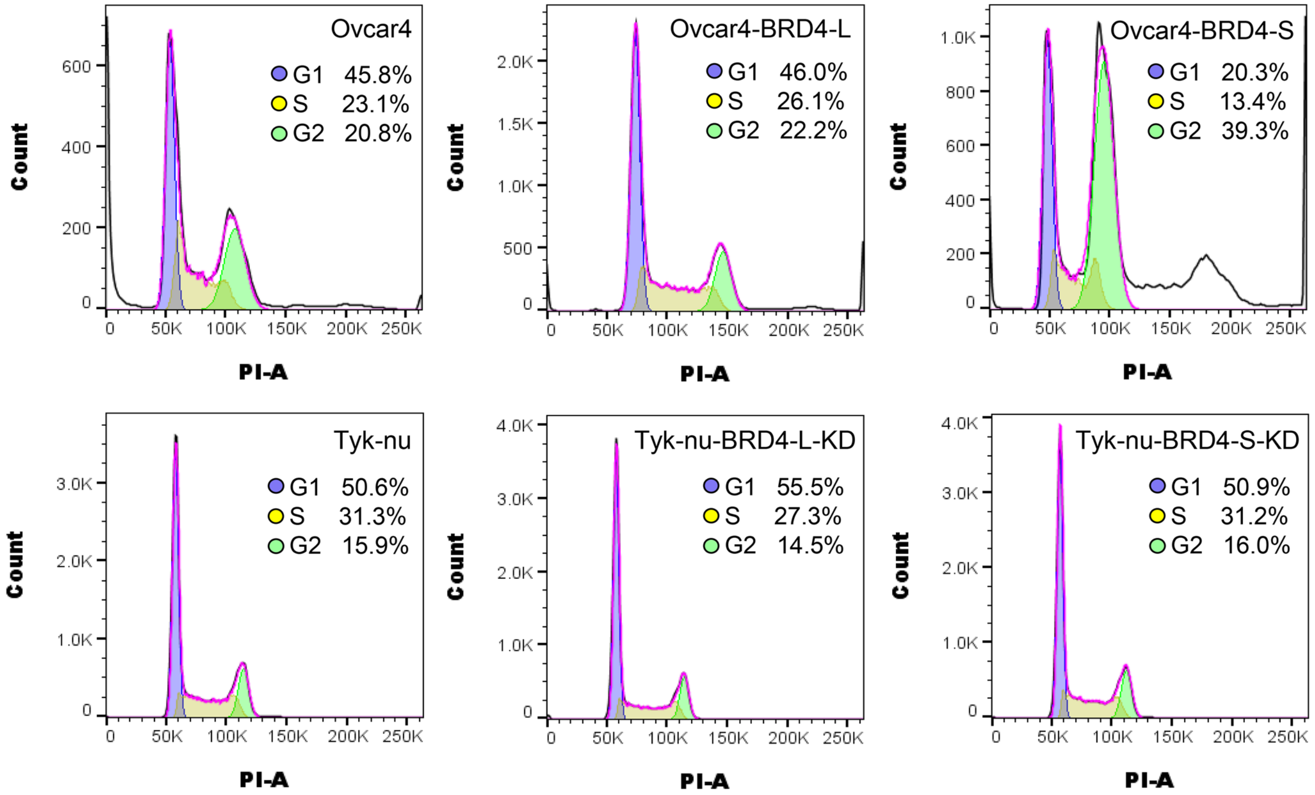

Supplementary Figure 5: Flow cytometry analysis of cell cycle distribution in Ovcar4 and Tyk-nu cell lines following overexpression or knockdown of BRD4 isoforms. Cells were stained with Propidium Iodide (PI) dye, which intercalates into cellular DNA and is directly proportional to DNA content. The representative histograms show the percentage of cells in each phase of the cell cycle. Data were analyzed and visualized by FlowJo v10.8.1 software.

Supplementary Table 1: Clinical-pathological characteristics of patients from whom PDX models have been derived

PDX ID	Age	Tumor stage	Disease status	OS (months)	BRCA status
PDX0003	44	IIIC	DOD	47.8	Negative
PDX0021	75	IIIC	DOD	18	N/A
PDX0027	66	IIIC	DOC	12	BRCA2
PDX0038	57	IIIC	DOD	14	N/A
PDX0059	68	IIIC	DOD	16	Negative
PDX0083	78	IIIC	DOD	37.5	Negative
PDX0100	61	IVB	DOD	25	Negative
PDX0110	63	IIIC	DOD	24.5	Negative
PDX0113	58	IIIC	DOD	19	Negative

Abbreviations: OS: overall survival; DOD: died of disease; DOC: died from other cause; N/A indicates that the respective status is unknown.

Supplementary Table 2: BRD4 transcripts counts in ovarian PDX models

Transcript ID	Length	Effective Length	PDX-0003	PDX-0021	PDX-0027	PDX-0038	PDX-0059	PDX-0083	PDX-0100	PDX-0110	PDX-0113
ENST00000679869.1	7231	6981	0.0	0.0	0.0	0.0	0.0	0.4	0.0	0.0	0.0
ENST00000263377.6 (BRD4-L)	7169	6919	7.4	4.8	7.1	4.0	6.2	6.6	6.1	3.8	5.2
ENST00000371835.8 (BRD4-S)	4624	4374	2.2	2.8	4.9	2.7	2.9	4.9	4.0	2.3	3.9
ENST00000360016.9	3240	2990	0.0	0.0	0.0	0.0	0.2	0.0	0.0	0.3	0.0
ENST00000594841.5	1821	1571	0.0	0.0	0.0	0.0	0.0	0.0	0.0	0.0	0.0
ENST00000601941.1	604	354	0.0	0.0	0.0	0.0	0.0	0.0	0.0	0.0	0.0
**Total TPM for BRD4**			9.6	7.6	12.0	6.7	9.3	11.9	10.1	6.4	9.1
**PSI (percentage of spliced-in) - BRD4-L**			76.6	62.8	59.0	59.9	66.3	55.5	60.4	59.1	56.8
**PSI - BRD4-S**			23.4	37.2	40.6	40.1	31.5	41.3	39.6	36.5	43.2
**BRD4-L/BRD4-S splicing ratio**			3.3	1.7	1.5	1.5	2.1	1.3	1.5	1.6	1.3

Percentage of spliced-in (PSI) index was calculated based on the total counts of all BRD4 isoforms. PSI values were used to estimate BRD4-L/BRD4-S splicing ratio. Transcript counts are normalized counts expressed in transcripts per million (TPM), and were determined using Salmon in Galaxy (usegalay.org). Transcripts encoding BRD4 isoform long or short are shown in the table.

Supplementary Table 3: A list of primers used to generate BRD4 overexpression or knockdown plasmids and primers used for qPCR experiments

Oligonucleotides	Source	Identifier
Cold fusion cloning BRD4-S forward primer: GGTGTCGTGACGCGGGCATGTCTGCGGAGAGCGG	This paper	CF-BRD4-S_pLV-F
Cold fusion cloning BRD4-S reverse primer: CCATGGTGGCGGATCTTAGGCAGGACCTGTTTCGGAG	This paper	CF-BRD4-S_pLV-R
Cold fusion cloning BRD4-L forward primer: GGTGTCGTGACGCGGCTATGTCTGCGGAGAGCGG	(Drumond-Bock et al., 2022)	CF-BRD4-L_pLV-F
Cold fusion cloning BRD4-L reverse primer: CCATGGTGGCGGATCCCTAGGTGCGCTCAGAAAAGA	(Drumond-Bock et al., 2022)	CF-BRD4-L_pLV-R
shRNA oligo for BRD4-S-KD forward primer: CCGGATTGGACACGGACTCTTAATACTCGAGTATTAAGAGTCCGTGTCCA ATTTTTG	This paper	F-BRD4-S-TRC9782
shRNA oligo for BRD4-S-KD reverse primer: AATTCAAAAAATTGGACACGGACTCTTAATACTCGAGTATTAAGAGTCCGT GTCCAAT	This paper	R-BRD4-S-TRC9782
shRNA oligo for BRD4-L-KD forward primer: CCGGCAGAGTGATCTATTGTCAATACTCGAGTATTGACAATAGATCACTC TGTTTTTG	This paper	F-BRD4-L-TRC5294
shRNA oligo for BRD4-L-KD reverse primer: AATTCAAAAACAGAGTGATCTATTGTCAATACTCGAGTATTGACAATAGAT CACTCTG	This paper	R-BRD4-L-TRC5294
qPCR forward primer for all BRD4 isoforms: ACCTCCAACCCTAACAAGCC	This paper	qPCR_BRD4_All_F
qPCR reverse primer for all BRD4 isoforms: TTTCCATAGTGTCTTGAGCACC	This paper	qPCR_BRD4_All_R
qPCR forward primer for endogenous BRD4 isoform long: AAGCCATGGCAGCTACCATTG	This paper	qPCR_BRD4-L_F
qPCR reverse primer for endogenous BRD4 isoform long: CAAAGTCAGAAGCCACCTAGG	This paper	qPCR_BRD4-L_R
qPCR forward primer for endogenous BRD4 isoform short: CCTAATCATTGGACACGGAC	This paper	qPCR_BRD4_S_F
qPCR reverse primer for endogenous BRD4 isoform short: TCCTGTCCCTTTCACGGAAGA	This paper	qPCR_BRD4_S_R
qPCR forward primer for human actin B: TGACCCAGATCATGTTTGAGACC	This paper	hACTB_qPCR-F
qPCR reverse primer for human actin B: GTCCAGACGCAGGATGGCATT	This paper	hACTB_qPCR-R

All sequences used to generate BRD4-S refer specifically to BRD4-S(a) isoform.

Supplementary Table 4: A list of antibodies used for Wes (ProteinSimple) immunoassay and for immunofluorescence staining

Antibodies	Source	Identifier
Rabbit monoclonal anti-BRD4	Abcam	Cat# ab128874; RRID:AB_11145462
Rabbit monoclonal anti-BRD4-L isoform (BRD4-L specific)	Cell Signaling	Cat# 13440; RRID:AB_2687578
Rabbit monoclonal anti-Vinculin	Cell Signaling	Cat# 13901; RRID:AB_2728768
Rabbit polyclonal anti-GAPDH	Santa Cruz Biotechnology	Cat# sc-25778; RRID:AB_10167668
Rabbit monoclonal anti-Phospho-H2A.X (Ser139)	Cell Signaling	Cat# 9718; RRID:AB_2118009
Anti-rabbit IgG (H+L), F(ab')2 fragment (Alexa Fluor 594 conjugate)	Cell Signaling	Cat# 8889; RRID:AB_2716249
Mouse monoclonal anti-Alpha-Tubulin (Alexa Fluor 488 conjugate)	Cell Signaling	Cat# 8058
